# The Gait Pattern Classification System for Children with Spastic Cerebral Palsy (GaP-CP)—A Validity Study

**DOI:** 10.3390/children12030269

**Published:** 2025-02-23

**Authors:** Eirini Papageorgiou, Laure Everaert, Ines Vandekerckhove, Britta Hanssen, Anja Van Campenhout, Els Ortibus, Kaat Desloovere

**Affiliations:** 1 Department of Rehabilitation Sciences, KU Leuven, 3001 Leuven, Belgium; eirini.papageorgiou@kuleuven.be (E.P.); laure.everaert@kuleuven.be (L.E.); ines.vandekerckhove@kuleuven.be (I.V.); britta.hanssen@kuleuven.be (B.H.); 2 Department of Physical Medicine and Rehabilitation, University Hospital Leuven, 3000 Leuven, Belgium; 3 Department of Development and Regeneration, Organ Systems, Locomotor and Neurological Disorders, KU Leuven, 3000 Leuven, Belgium; anja.vancampenhout@uzleuven.be (A.V.C.); els.ortibus@uzleuven.be (E.O.); 4 Department of Orthopedics, University Hospital Leuven, 3000 Leuven, Belgium; 5 Center for Developmental Disabilities, 3000 Leuven, Belgium; 6 Clinical Motion Analysis Laboratory, University Hospital Leuven, 3212 Pellenberg, Belgium

**Keywords:** spastic cerebral palsy, gait classification system, content validity, construct validity, classification rules, patient-related characteristics, clinical impairment scores

## Abstract

Background: In order to classify the gait of children with spastic cerebral palsy (sCP), the “gait pattern classification system for children with sCP” (GaP-CP) has been developed, based on a systematic review and complemented by an additional class of “mild deviations”. The objective of the current study was to examine the content and construct validity of the GaP-CP. Methods: Statistical non-parametric comparisons identified the differences between the kinematics (N = 270) and kinetics (N = 208) of children with sCP and children with typically developing gait (N = 56) (content validity—aim 1), and between neighboring patterns (construct validity—aim 2a). Pattern-specific clinical phenotypes were explored based on the differences between all gait patterns (Kruskal–Wallis comparisons with post hoc Mann–Whitney U tests) in patient- and impairment-specific characteristics (construct validity—aim 2b). Results: The apparent and true equinus were the most and least prevalent patterns, respectively. The majority of the original classification rules were confirmed by the patient data, indicating good content validity of the GaP-CP. Its construct validity was strengthened by distinguishing between gait deviations of neighboring patterns and differentiating between gait patterns based on patient-specific characteristics, leading to pattern-specific phenotypes. Yet, pattern-specific phenotypes could not be clearly defined based on the impairment-specific comparisons. Conclusions: The current results support the validity of the GaP-CP. Refined classification rules, using more precise terminology, establish a roadmap for its clinical implementation. The pattern-specific clinical phenotypes enable its use in a clinical context and may aid clinical decision-making.

## 1. Introduction

Gait deviations in ambulant children with spastic cerebral palsy (sCP) are usually evaluated through three-dimensional gait analysis (3DGA) to support and improve treatment planning [1]. To facilitate the interpretation of extensive 3DGA datasets and the communication between healthcare practitioners, various gait classification systems (GCSs) have been suggested [2,3]. Previous classification systems mostly focused on sagittal plane kinematic deviations. Moreover, reliability or validation studies were scarcely performed and were conducted long after the original publications [2,3]. These methodological shortcomings, as well as the wide heterogeneity in pattern definitions, may have hindered the clinical applicability of previous GCSs.

A systematic literature review summarized the most commonly used gait patterns, involving frequently observed lower limb motion combinations in children with sCP. It resulted in an overview of six gait patterns, i.e., dropfoot, genu recurvatum, true equinus, jump gait, apparent equinus and crouch gait, describing kinematic deviations in the sagittal plane [3]. These six gait patterns were chosen from a panel of four reviewers, based on three criteria: adequate pattern definitions, patterns showing “good” or “excellent” clinical applicability, and patterns that have been used by at least two independent research groups after the original publications were introduced. These patterns, stemming from various GCSs, have previously been shown to be reliable [3]. In recent years, they have also been applied by different research groups to several samples of patients with sCP [4,5,6,7], demonstrating strong clinical and research applicability. However, this overview of six gait patterns did not include a class for “mild deviations” characterized by minor deviations across all lower limb segments or joints, as previously proposed [8,9,10]. With this addition, the “gait pattern classification system for children with sCP” (GaP-CP) was compiled. However, the original definitions of the gait patterns comprising the GaP-CP have not yet been validated using one extensive sample. Similarly, the kinetics or out-of-sagittal-plane motions (i.e., motions in the coronal and transverse planes) of these patterns have not yet been described, nor studied in the past [3]. Understanding the biomechanical mechanisms underlying kinematic outputs (i.e., kinetics) and the pattern-specific gait characteristics in the other planes of motion could not only refine classification rules, but also enhance the clinical decision-making process, and could provide more precise pattern-specific “fingerprints”.

Moreover, confusion can arise during classification, as pattern definitions vary greatly among studies [3,11], especially between “neighboring patterns”, i.e., patterns that share similar gait deviations. For instance, jump gait, apparent equinus and crouch gait share the feature of increased knee flexion and should be distinguished based on the different ankle joint positions [10,12]. Yet, a more recent systematic review on flexed-knee gait concluded that crouch gait refers to increased knee flexion during the stance, irrespective of the ankle joint position [11]. Such contradictory criteria demand improved validity and clear classification rules, facilitating the distinction of the most commonly described gait patterns in children with sCP.

In addition, when a new GCS is introduced, it is important to describe the patient-related characteristics or concomitant impairments of the considered gait patterns. Even though several gait patterns were originally defined for specific use in children with either unilateral or bilateral sCP (usCP and bsCP, respectively) [10,13,14], they have been observed in both populations [3,15,16]. Furthermore, male sex was found to be a higher risk factor for cerebral palsy [17], but it is uncertain whether the gait patterns of the GaP-CP are sex-specific. Increasing age, previous treatments, or higher levels on the gross motor function classification system (GMFCS) have also been described for certain patterns (e.g., crouch gait) [18,19]. Yet, a detailed pattern-specific description of the patient-related characteristics is still missing. In the same line, the clinical validity of a GCS would increase when the pattern-specific clinical impairments, such as spasticity, muscle strength, selectivity, and contractures (i.e., passive range of motion—pROM), would be known. In general, more severe clinical impairments have been described for patients with more gait deviations [19,20,21]. Information on the clinical presentation of the GaP-CP gait patterns may facilitate the integration and implementation of gait patterns into clinical decision-making, along with the appraisal of treatment outcomes [2,3] or the longitudinal inspection of patients’ gait [9].

Previous studies on the potential relationships between sCP gait and clinical impairments have shown contradictory results [20,21,22,23,24], with causal relationships between clinical impairments and gait deviations being difficult to establish [25]. Especially, the relationship between gait and spasticity has been questioned [24,26]. This is remarkable given the fact that, from a clinical perspective, spasticity has been considered to contribute to various gait deviations [10,27,28], and is often treated. A detailed description of the clinical impairments of the GaP-CP gait patterns and the differences between them is still lacking. It is already known how the GaP-CP patterns respond to spasticity reduction treatments, including changes in both kinematics and clinical impairment scores [16,29]. Due to the broad spectrum of commonly used clinical impairment scores, data reduction may be needed, which could be performed by employing composite impairment scores. These composite scores provide an overall description of the patients’ neuromusculoskeletal symptoms [23,30,31]. On the other hand, muscle-specific impairments illustrate a detailed view of patients’ neuromuscular problems [23].

**Figure 1:**
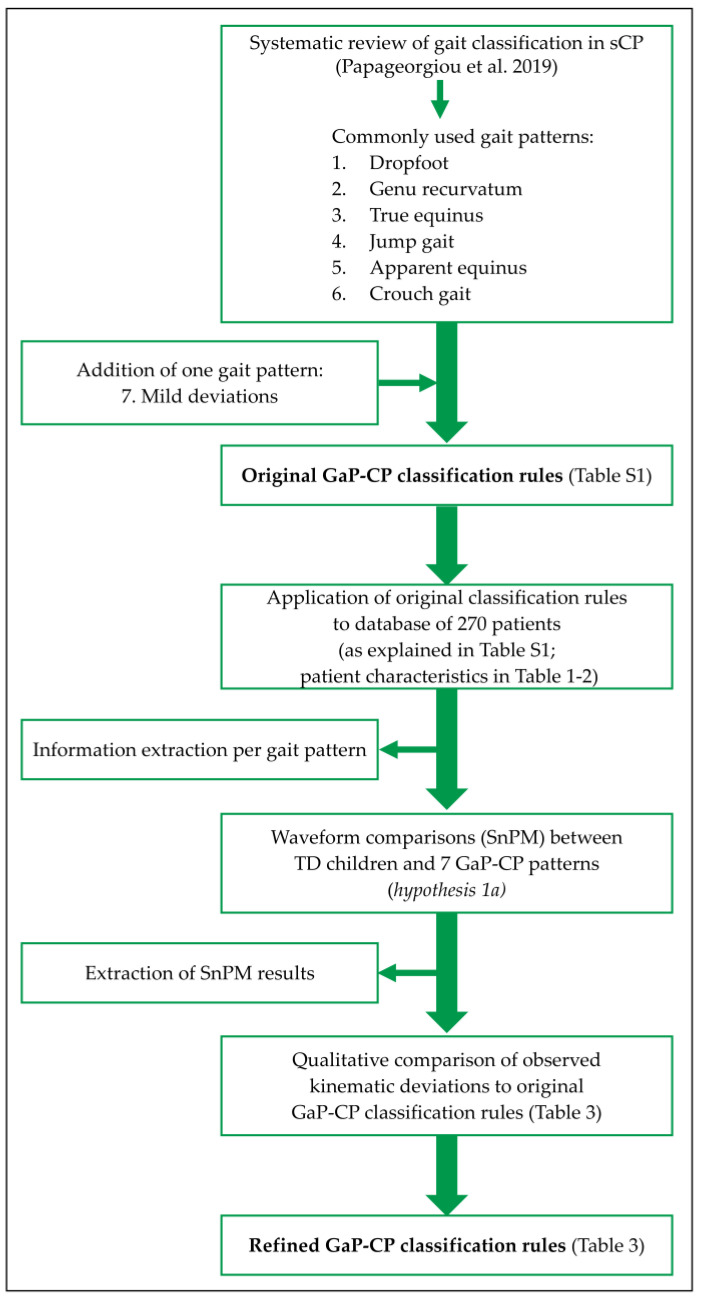
The workflow followed for hypothesis 1a: original gait pattern definitions can be confirmed within an extensive dataset. Abbreviations: sCP = spastic cerebral palsy; GaP-CP = gait pattern classification system for children with sCP [3]; SnPM = statistical non-parametric mapping; TD = typically developing.

The validity and clinical applicability of the GaP-CP should be further defined. Firstly, its content validity should be investigated following a data-driven approach, i.e., analyzing an extensive dataset, to prove that it truly measures what it should [32] and describes all the patterns in sCP [3] (aim 1). Thereby, the GaP-CP should be able to identify the reported deviations from typically developing (TD) gait (hypothesis 1a: original gait pattern definitions can be confirmed within an extensive dataset). Moreover, the description of GaP-CP should preferably also include additional information based on the concurrent kinetic and out-of-sagittal-plane deviations which have not been studied previously, as well as some more detailed kinematic features than originally described. These are here labeled as “fingerprints” (hypothesis 1b: pattern-specific “fingerprints“ are present). Furthermore, its construct validity should be established (aim 2). One aspect of construct validity in the current context is the issue of verifying gait-specific differences between gait patterns that are considered clinically relevant (e.g., neighboring gait patterns) [32] (hypothesis 2a: neighboring gait patterns can be differentiated based on their continuous gait waveforms). In addition, construct validity can evaluate whether patient-specific characteristics or clinical impairment scores are able to discriminate among groups that are theoretically known to be distinct [33] (hypothesis 2b: the gait patterns can be sufficiently differentiated from each other based on their patient- or impairment-specific characteristics). In the end, all study results can be combined to refine the classification rules for each gait pattern, providing clear guidelines as a roadmap for using the GaP-CP. The workflow that was followed for hypothesis 1a, starting from the composition of the GaP-CP to the refinement of its classification rules, is displayed in Figure 1. This study’s innovation lies in integrating gait-, patient- and impairment-specific differences across all GaP-CP gait patterns. These differences help define pattern-specific clinical phenotypes, enhancing the GaP-CP’s clinical applicability and its role in clinical decision-making. Additionally, incorporating gait patterns from various previously described GCSs makes the GaP-CP a more cost-effective tool, as clinicians and researchers need to familiarize themselves with only one comprehensive GCS, which consolidates all relevant information and applies to a broader range of children with sCP.

## 2. Materials and Methods

### 2.1. Subjects

A retrospective sample of patients with sCP was selected from the database of the Clinical Motion Analysis Laboratory of University Hospital Leuven. Permission to access and process pseudo-anonymized sCP patient data, acquired during standard medical care, was granted by the respective Medical Ethical Committee (Ethical Committee UZ Leuven/KU Leuven; S56036).

The inclusion criteria were as follows: (i) predominantly sCP, i.e., without marked signs of ataxia or dyskinesia; (ii) usCP or bsCP; (iii) ambulatory capacity (GMFCS levels I-III); (iv) ages between 3 and 18 years at the time of 3DGA. One session per patient was chosen based on the quality of the sagittal plane kinematics, as well as the availability of complete clinical examination information. If two 3DGA sessions of one patient fulfilled these criteria, the earliest session was included, to minimize the effect of any potential treatment(s) or growth on the gait of the considered patients. A total of 270 3DGA sessions from children with sCP were considered, of which 208 contained good-quality kinetic data. Reference gait data of 56 TD children were used, aged between 3 and 18 years, without previous neuromotor or musculoskeletal disorders.

### 2.2. Data Collection and Processing

All 3DGAs were acquired at a self-selected walking speed in barefoot conditions. Retroreflective markers with a 14 mm diameter were placed on bony landmarks on the skin based on the lower body Vicon Plug-In-Gait marker model (Vicon, Oxford, UK) [34,35]. A Vicon system containing 10 to 15 optoelectronic cameras (Vicon-UK, Oxford, UK) captured the marker trajectories, sampled at 100 Hz, and two force plates—embedded in a 10 m walkway—recorded the ground reaction forces at 1500 Hz (Advanced Mechanical Technology Inc., Watertown, MA, USA). Gait cycles were identified based on the force plate data, when available, and/or on the ankle and foot motion. Due to the retrospective nature of the study, the data were captured with various versions of the Nexus software throughout the years (Nexus 1.5-2.10, Vicon, Oxford, UK).

The kinematic and kinetic waveforms were time-normalized to the gait cycle, representing 100%, and the joint moments and power were normalized to body mass. The quality of the gait cycles and the presence of artifacts or outliers were visually evaluated in a custom-made Matlab^®^ script (The MathWorks, Natick, MA, USA, version 2019). Thereby, all available gait cycles of each lower limb were simultaneously shown, along with the average kinematic waveform. The latter was considered as a reference, and gait cycles were excluded in cases where they displayed increased variability from the average waveform (≥2 standard deviations). Such trials were excluded to ensure that only representative gait cycles were retained, depicting the usual gait pattern of each individual. Moreover, gait cycles were excluded when the knee varus–valgus ROM was larger than or equal to 15° and/or a knee valgus angle of 10° or more during swing was present. Such values would point towards a higher risk of marker misplacement. Kinetic gait cycles were excluded in cases of observed artifacts (such as incomplete hitting of the force plate or bilateral force plate contact). The TD gait data were collected in the same laboratory, using the same methods and were plotted for a visual comparison to the sCP gait patterns. Kinematic gait cycles were classified into one of the seven gait patterns of the GaP-CP by a clinician with >5 years of experience with gait classification, following the procedure described in Table S1. For the purposes of a previous study, this clinician had classified 196 gait trials twice, showing a substantial intra-rater reliability (k = 0.76, confidence interval: 0.65–0.87) [36]. Gait cycles that could not be classified based on the available definitions were labeled as “non-classifiable” and were excluded from all analyses. Thereafter, the average kinematic and kinetic gait cycles of the affected lower limb of children with usCP and of the most spastic and/or weakest lower limb based on the clinical examination of bsCP children were selected.

On the day of the 3DGA, during the standard clinical examination procedure, patient-specific characteristics (i.e., age, topographic classification, sex, and GMFCS level), along with the clinical impairments of spasticity, strength, selectivity, and pROM of the lower limb muscles acting in the sagittal plane were collected, similar to the procedure described in [23]. At first, muscle-specific impairment scores were collected. Subsequently, these scores were summed to form joint-specific scores, and the latter were summed to create composite impairment scores (as specified in more detail below). This study focused on the composite and the muscle-specific impairment scores.

Spasticity was measured with the Modified Ashworth Scale (MAS) [37] for the hip flexors, hamstrings, rectus femoris, gastrocnemius, and soleus muscles (ordinal scale, range: 0–4). Composite spasticity was calculated as the sum of the joint spasticity scores, namely the spasticity of the hip flexors (hip spasticity), the sum of the hamstrings and rectus femoris (knee spasticity), and the median of the MAS scores of the ankle plantar flexors (ankle spasticity), and ranged from 0 to 16.

The strength of the hip and knee flexors and extensors, ankle dorsiflexors, with both the knee extended and in 90° flexion, as well as the ankle plantar flexors, were measured with the manual muscle testing scale (MMT) [38] (ordinal scale, range: 0–5). The composite strength score was defined as the sum of the three joint strength scores, namely hip strength (sum of hip flexors and extensors), knee strength (sum of flexors and extensors) and ankle strength (sum of plantar flexors and median of ankle dorsiflexors), ranging from 0 to 30.

Selectivity was measured by a scale proposed by Trost et al. [39] (ordinal scale), focusing on the same muscles as for the MMT measurements, ranging from 0 to 2 for the muscle-specific selectivity scores and from 0 to 12 for the composite selectivity score (which represented the sum of the selectivity scores for the hip and knee flexors and extensors and the ankle plantar and dorsiflexors).

Finally, pROM was measured using a goniometer. Only motions that are often impaired for children with sCP were considered, i.e., hip extension (deficit), using the Thomas test; hamstring length, using the unilateral and bilateral popliteal angles; and ankle dorsiflexion, with both the knee extended and in 90° flexion. The bilateral popliteal angle refers to the true hamstring length and is measured with the contralateral hip in flexion, while the unilateral popliteal angle is measured with the contralateral hip in extension, and may thus be influenced by the pelvic position and involvement of the psoas [40]. All goniometric measurements were transformed to a 3-point ordinal scale (range: 0–2), using the 25th and 75th percentiles of the entire sample of children with sCP as cut-offs, whereby values higher than the 75th percentile indicated no or slight contractures (score of “0”), values between the 25th and 75th percentiles showed moderate contractures (score of “1”) and values lower than the 25th percentile indicated severe contractures (score of “2”). The composite pROM score was calculated based on the sum of the joint contracture scores, namely the hip pROM (hip extension), the knee pROM (median of the unilateral and bilateral popliteal angles) and the ankle pROM (median of the ankle dorsiflexion measurements), ranging from 0 to 6.

Higher (composite) spasticity and pROM scores indicated higher impairments, while higher (composite) strength and selectivity scores indicated a better potential for the patients, i.e., lower impairments.

### 2.3. Data Analysis

Descriptive statistics were utilized for the sample characteristics for both children with sCP and TD children. Based on the Shapiro–Wilk test, the majority of the data were not normally distributed; therefore, medians and interquartile ranges were used to describe continuous variables (IBM SPSS Statistics for Windows, version 24—IBM Corp., Armonk, NY, USA).

For hypotheses 1a, 1b, and 2a, statistical parametric mapping (SPM—SPM1d version 0.4.7, http://www.spm1d.org/, accessed on 12 February2022) in Matlab^®^ (The MathWorks, Natick, MA, USA, version 2019) was employed [41]. SPM was chosen because it allows hypothesis testing across the entire continuous waveforms. It employs Random Field Theory, enabling the calculation of critical thresholds that can be exceeded in alpha (α) % of the experiments, i.e., in case of statistically significant differences. In this case, suprathreshold clusters are formed, displaying the regions within the gait cycle where the statistically significant differences are observed. These are often reported using the critical threshold that is crossed, the extent (or duration) of the clusters, their range (first and last points), and their *p*-values. The continuous gait waveforms were not normally distributed, based on the built-in SPM function; hence, the non-parametric version (SnPM) and the respective test equivalents were used, with 10,000 iterations.

All analyses were performed on the vector level (i.e., combining all lower limb segments or joints), taking the vector component covariance into account. If significance was achieved, post hoc segment- or joint-level comparisons were conducted [42]. Bonferroni corrections were applied where needed to account for multiple testing (i.e., number of gait patterns, motions or pairs).

For the content validity (hypotheses 1a and 1b), kinematic data across all three motion planes and the sagittal plane kinetics of each of the seven gait patterns were compared to TD gait using non-parametric two-sample Hotelling’s T^2^ tests, with α = 0.007 for each vector. Post hoc comparisons (non-parametric two-sample *t*-tests) focused on the motions of the pelvis, hip, knee, and ankle in the sagittal plane (α = 0.002), the sagittal plane moments or powers of the hip, knee, and ankle (α = 0.002), the coronal plane motions of the pelvis and hip (α = 0.0035) and the transverse plane motions of the pelvis and hip, as well as the foot progression angle (α = 0.002). Since the dropfoot and apparent equinus patterns also included definitions of discrete variables, namely showing adequate ankle ROM in stance, these were compared between either of the two gait patterns and the TD sample using Mann–Whitney U tests (α = 0.05) in SPSS.

For the first part of the construct validity (hypothesis 2a), among groups, vector-level comparisons were conducted (non-parametric multivariate analysis of variance—MANOVA, α = 0.05), with post hoc pairwise comparisons (N = 6 pairs) at the vector level (non-parametric two-sample Hotelling’s T^2^ tests, with α = 0.008) and at the segment or joint level (non-parametric two-sample *t*-tests, all α = 0.002, apart from the pelvis and hip in the coronal plane, where α = 0.004). Despite some shared gait features, neighboring patterns have been used as separate clinical gait representations. It was, for example, meaningful to compare the continuous waveforms of genu recurvatum to true equinus because of their shared gait features of knee extension and ankle plantarflexion. Because such neighboring patterns are especially challenging to distinguish, pairwise comparisons highlighted key discriminating gait features. The continuous waveforms of gait patterns that are fundamentally different from each other (such as genu recurvatum and crouch gait) were not statistically compared.

Following the approach applied in previous studies [16,29], the suprathreshold clusters of each segment or joint were deemed relevant if they displayed a minimal cluster duration of ≥3% of the gait cycle [23] and if the differences in means between the compared continuous waveforms exceeded the respective standard errors of measurement (SEMs) [43] for at least 80% of the duration of the suprathreshold clusters. For the kinetics, only clusters identified during the stance phase were judged based on the SEMs [43] and were further discussed in this study.

For the second part of the construct validity (hypothesis 2b), the distribution of the patient-specific characteristics among the patterns was examined using SPSS. Chi-squared (χ^2^) tests were used for categorical variables (topographic classification, gender, GMFCS levels), and Kruskal–Wallis tests were used for continuous variables (α = 0.05). In case the latter were statistically significant, post hoc Mann–Whitney U tests were employed and Bonferroni correction was again applied (α = 0.007). Finally, effect sizes (Cohen’s d) [44] were calculated online (https://www.psychometrica.de/effect_size.html—section 11, accessed on 16 May 2022) for all statistically significant Mann–Whitney U test comparisons.

## 3. Results

The general characteristics for TD children and patients with sCP are reported in Table 1, including, but not limited to, information regarding the amount of kinematic and kinetic trials available, ages at the time of 3DGA, topographic classification, sex, and GMFCS levels. The total sample of 270 children with sCP had a median age of 7.7 years (range 3.6–17.6 years); 45.5% with usCP and 54.5% with bsCP; 59.3% boys and 40.7% girls; and 58.1%, 30.4%, and 11.5% with GMFCS I, II and III, respectively. A left-side involvement was found in 59 (48%) of children with usCP. Based on the clinical examination, the left lower limb was characterized by the highest levels of spasticity and/or weakness in 65 (44%) children with bsCP and was subsequently analyzed. The most prevalent pattern was apparent equinus (N = 71) and the least prevalent was true equinus (N = 17). The distributions of both the composite and muscle-specific clinical impairment scores (expressed in medians and interquartile ranges) are reported in Table 2, along with the *p*-values resulting from the Kruskal–Wallis comparisons, showing that differences among patterns were observed for all clinical impairment scores.

### 3.1. Hypothesis 1a: Original Gait Pattern Definitions Can Be Confirmed Within an Extensive Dataset

**Figure 2:**
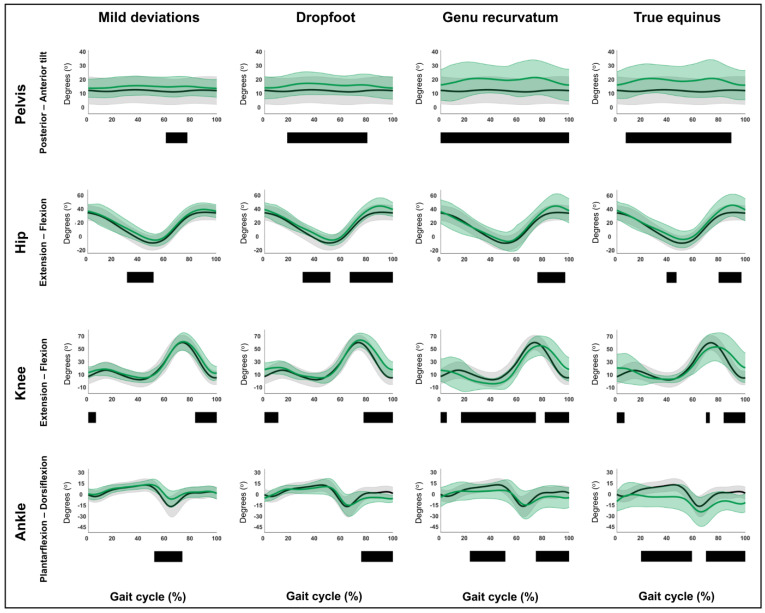
Hypothesis 1a: original gait pattern definitions can be confirmed within an extensive dataset. From left to right: the sagittal plane kinematics of the mild deviations, dropfoot, genu recurvatum, and true equinus patterns compared to those of typically developing children. From top to bottom: the motions of the pelvis, hip, knee, and ankle for each of these gait patterns throughout the gait cycle [*x*-axis, 0–100%; *y*-axis, degrees of motion (^o^)] are shown in green (mean ± 1 standard deviation), while the respective kinematics of typically developing children are shown in gray (mean ± 1 standard deviation). The results of the post hoc, statistical non-parametric comparisons (non-parametric two-sample *t*-tests) throughout the gait cycle are summarized using black bars for the statistically significant differences (i.e., suprathreshold clusters), all *p* ≤ 0.001. The details of each cluster are shown in Table S2.

**Figure 3:**
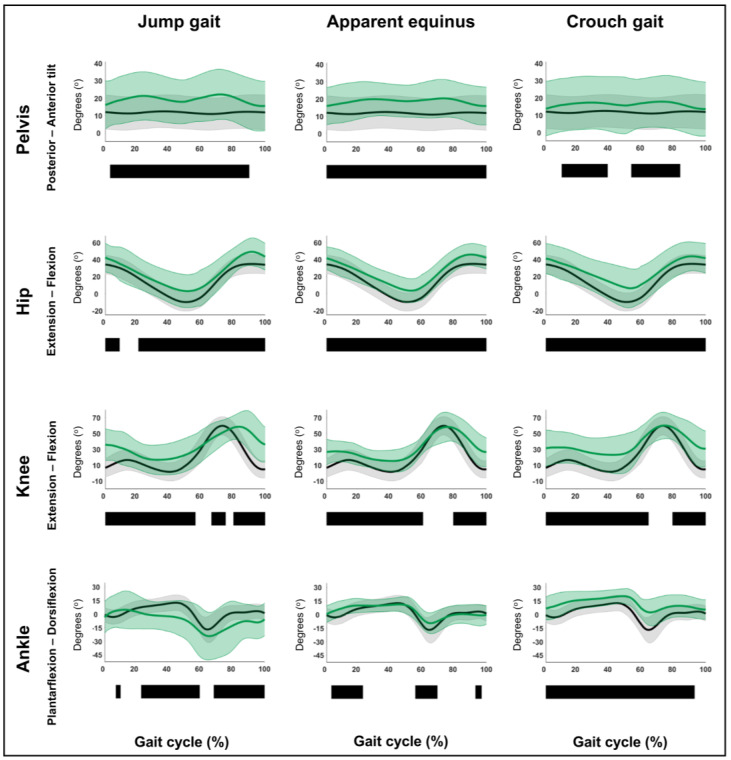
Hypothesis 1a: original gait pattern definitions can be confirmed within an extensive dataset. From left to right: the sagittal plane kinematics of the jump gait, apparent equinus, and crouch gait patterns compared to those of typically developing children. From top to bottom: the motions of the pelvis, hip, knee, and ankle for each of these gait patterns throughout the gait cycle [*x*-axis, 0–100%; *y*-axis, degrees of motion (^o^)] are shown in green (mean ± 1 standard deviation), while the respective kinematics of typically developing children are shown in gray (mean ± 1 standard deviation). The results of the post hoc, statistical non-parametric comparisons (non-parametric two-sample *t*-tests) throughout the gait cycle are summarized using black bars for the statistically significant differences (i.e., suprathreshold clusters), all *p* ≤ 0.001. The details of each cluster are shown in Table S2.

The detailed results regarding hypothesis 1a are reported in Figure 2 and Figure 3 and Table S2. The sagittal plane kinematic vectors of each pattern were statistically significantly different from TD gait. Kinematic differences were observed for all post hoc, motion-specific comparisons (*p* ≤ 0.001), whereby all identified suprathreshold clusters exceeded the respective SEMs for over 80% of their duration. In detail, Figure 2 and Figure 3 depict the four sagittal plane motions of the seven GaP-CP gait patterns in green (mean ± 1 standard deviation), compared to those of the TD children (in gray, mean ± 1 standard deviation) throughout the gait cycle. The SnPM results (i.e., suprathreshold clusters identifying statistically significant differences between each pattern and TD gait) are summarized using black bars.

Table 3 provides a detailed description of the original classification rules, next to the identified kinematic deviations from TD gait found in this study. These deviations were subsequently judged based on whether they confirmed the original classification rules or not. Additional information on clusters was provided, for example, regarding clusters spanning a larger part of the gait cycle than originally described. The SnPM comparisons of the continuous waveforms confirmed and refined the original classification rules for the majority of the patterns regarding their detailed characteristics, as shown in Table 3. However, the comparison of the ankle ROM during stance between TD gait and either children with dropfoot or apparent equinus revealed that in the current dataset, ankle ROM in stance (discrete variable) was diminished in both gait patterns (both *p* ≤ 0.001). Furthermore, the waveform-based comparisons revealed some additional prevalent kinematic deviations from TD gait, which are highlighted in Table S2. These do not constitute hard classification rules but indicate frequent pattern behavior and may be considered as “fingerprints” (see also hypothesis 1b below).

### 3.2. Hypothesis 1b: Pattern-Specific “Fingerprints” Are Present

**Figure 4:**
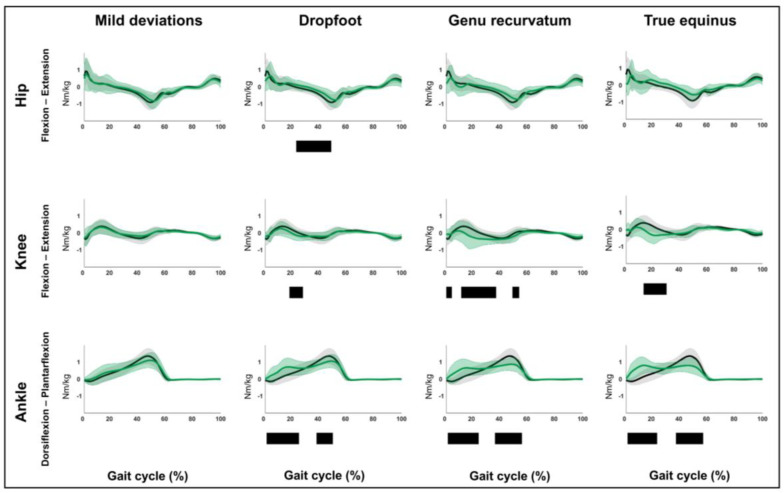
Hypothesis 1b: pattern-specific “fingerprints” are present. From left to right: the sagittal plane moments of the mild deviations, dropfoot, genu recurvatum, and true equinus patterns compared to those of typically developing children. From top to bottom: the moments of the hip, knee, and ankle for each of these gait patterns throughout the gait cycle [*x*-axis, 0–100%; *y*-axis, Nm/kg)] are shown in green (mean ± 1 standard deviation), while the respective moments of typically developing children are shown in gray (mean ± 1 standard deviation). The results of the post hoc, statistical non-parametric comparisons (non-parametric two-sample *t*-tests) throughout the gait cycle are summarized using black bars for the statistically significant differences (i.e., suprathreshold clusters), all *p* ≤ 0.001. The details of each cluster are shown in Table S3.

**Figure 5:**
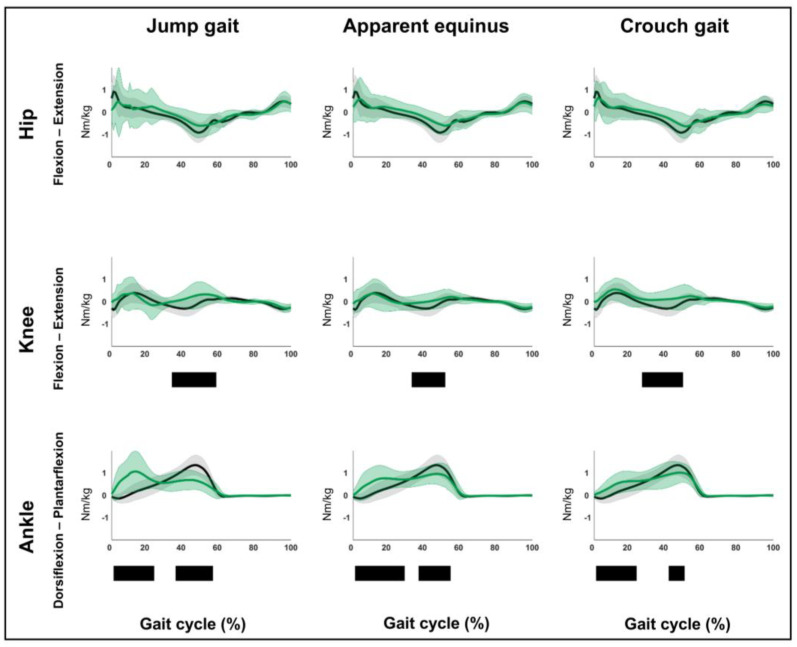
Hypothesis 1b: pattern-specific “fingerprints” are present. From left to right: the sagittal plane moments of the jump gait, apparent equinus, and crouch gait patterns compared to those of typically developing children. From top to bottom: the moments of the hip, knee, and ankle for each of these gait patterns throughout the gait cycle [*x*-axis, 0–100%; *y*-axis, Nm/kg)] are shown in green (mean ± 1 standard deviation), while the respective moments of typically developing children are shown in gray (mean ± 1 standard deviation). The results of the post hoc, statistical non-parametric comparisons (non-parametric two-sample *t*-tests) throughout the gait cycle are summarized using black bars for the statistically significant differences (i.e., suprathreshold clusters), all *p* ≤ 0.001. The details of each cluster are shown in Table S3.

**Figure 6:**
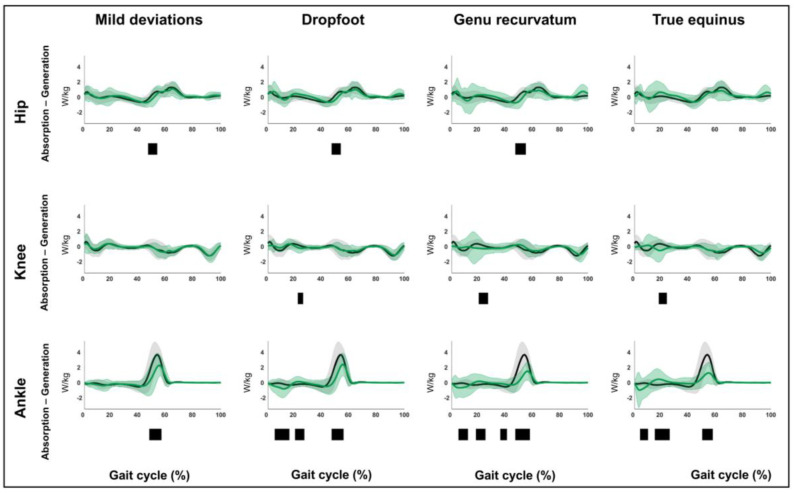
Hypothesis 1b: pattern-specific “fingerprints” are present. From left to right: the sagittal plane powers of the mild deviations, dropfoot, genu recurvatum, and true equinus patterns compared to those of typically developing children. From top to bottom: the powers of the hip, knee, and ankle for each of these gait patterns throughout the gait cycle [*x*-axis, 0–100%; *y*-axis, W/kg)] are shown in green (mean ± 1 standard deviation), while the respective powers of typically developing children are shown in gray (mean ± 1 standard deviation). The results of the post hoc, statistical non-parametric comparisons (non-parametric two-sample *t*-tests) throughout the gait cycle are summarized using black bars for the statistically significant differences (i.e., suprathreshold clusters), all *p* ≤ 0.001. The details of each cluster are shown in Table S3.

**Figure 7:**
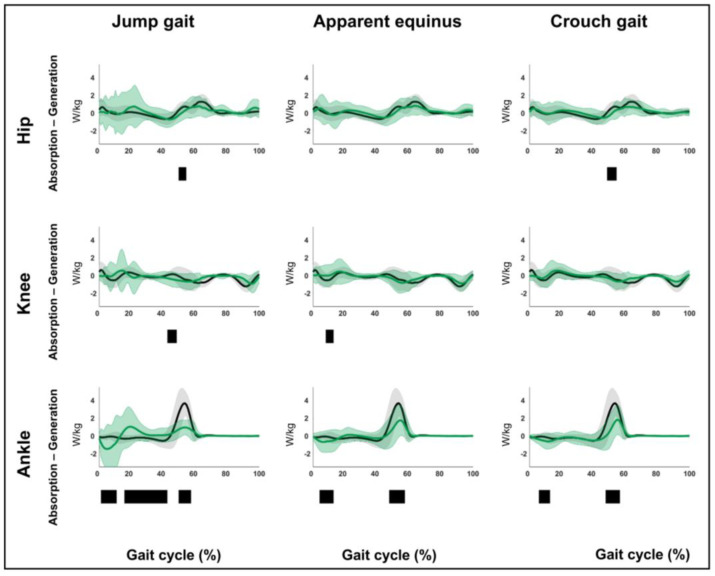
Hypothesis 1b: pattern-specific “fingerprints” are present. From left to right: the sagittal plane powers of the jump gait, apparent equinus, and crouch gait patterns compared to those of typically developing children. From top to bottom: the powers of the hip, knee, and ankle for each of these gait patterns throughout the gait cycle [*x*-axis, 0–100%; *y*-axis, W/kg)] are shown in green (mean ± 1 standard deviation), while the respective powers of typically developing children are shown in gray (mean ± 1 standard deviation). The results of the post hoc, statistical non-parametric comparisons (non-parametric two-sample *t*-tests) throughout the gait cycle are summarized using black bars for the statistically significant differences (i.e., suprathreshold clusters), all *p* ≤ 0.001. The details of each cluster are shown in Table S3.

**Figure 8:**
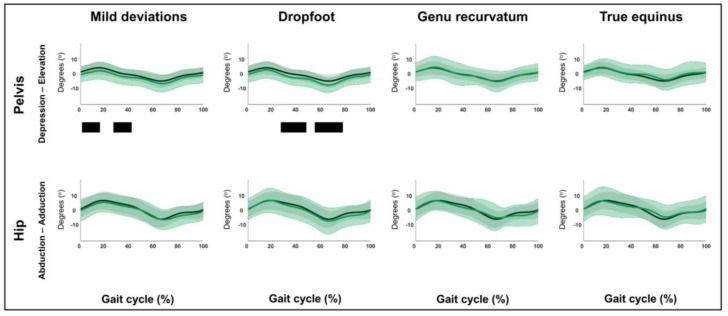
Hypothesis 1b: pattern-specific “fingerprints” are present. From left to right: the coronal plane motions of the mild deviations, dropfoot, genu recurvatum, and true equinus patterns compared to those of typically developing children. From top to bottom: the motions of the pelvis and hip for each of these gait patterns throughout the gait cycle [*x*-axis, 0–100%; *y*-axis, degrees of motion (^o^)] are shown in green (mean ± 1 standard deviation), while the respective motions of typically developing children are shown in gray (mean ± 1 standard deviation). The results of the post hoc, statistical non-parametric comparisons (non-parametric two-sample *t*-tests) throughout the gait cycle are summarized using black bars for the statistically significant differences (i.e., suprathreshold clusters), all *p* ≤ 0.001. The details of each cluster are shown in Table S4.

**Figure 9:**
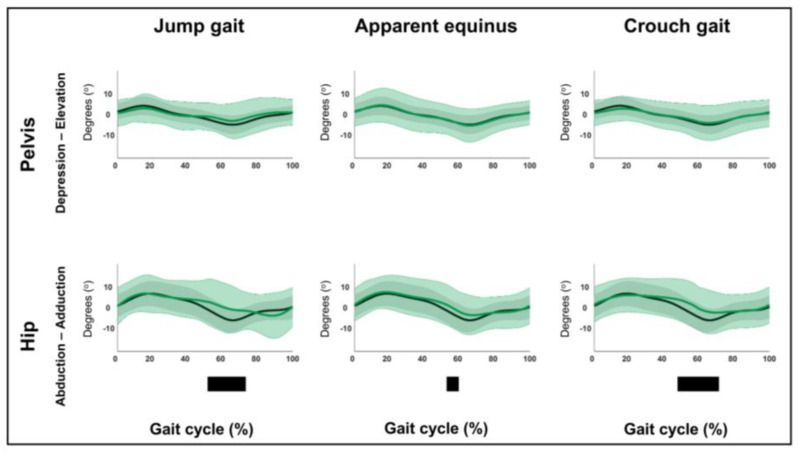
Hypothesis 1b: pattern-specific “fingerprints” are present. From left to right: the coronal plane motions of the jump gait, apparent equinus, and crouch gait patterns compared to those of typically developing children. From top to bottom: the motions of the pelvis and hip for each of these gait patterns throughout the gait cycle [*x*-axis, 0–100%; *y*-axis, degrees of motion (^o^)] are shown in green (mean ± 1 standard deviation), while the respective motions of typically developing children are shown in gray (mean ± 1 standard deviation). The results of the post hoc, statistical non-parametric comparisons (non-parametric two-sample *t*-tests) throughout the gait cycle are summarized using black bars for the statistically significant differences (i.e., suprathreshold clusters), all *p* ≤ 0.001. The details of each cluster are shown in Table S4.

**Figure 10:**
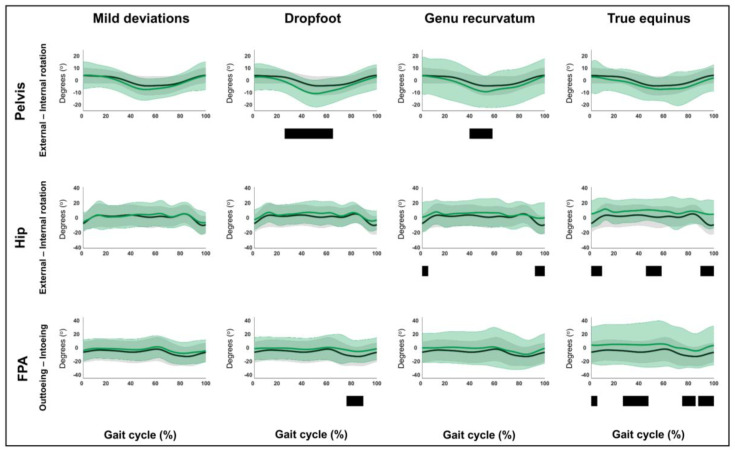
Hypothesis 1b: pattern-specific “fingerprints” are present. From left to right: the transverse plane motions of the mild deviations, dropfoot, genu recurvatum, and true equinus patterns compared to those of typically developing children. From top to bottom: the motions of the pelvis, hip, and foot progression angle (FPA) for each of these gait patterns throughout the gait cycle [*x*-axis, 0–100%; *y*-axis, degrees of motion (^o^)] are shown in green (mean ± 1 standard deviation), while the respective motions of typically developing children are shown in gray (mean ± 1 standard deviation). The results of the post hoc, statistical non-parametric comparisons (non-parametric two-sample *t*-tests) throughout the gait cycle are summarized using black bars for the statistically significant differences (i.e., suprathreshold clusters), all *p* ≤ 0.001. The details of each cluster are shown in Table S4.

**Figure 11:**
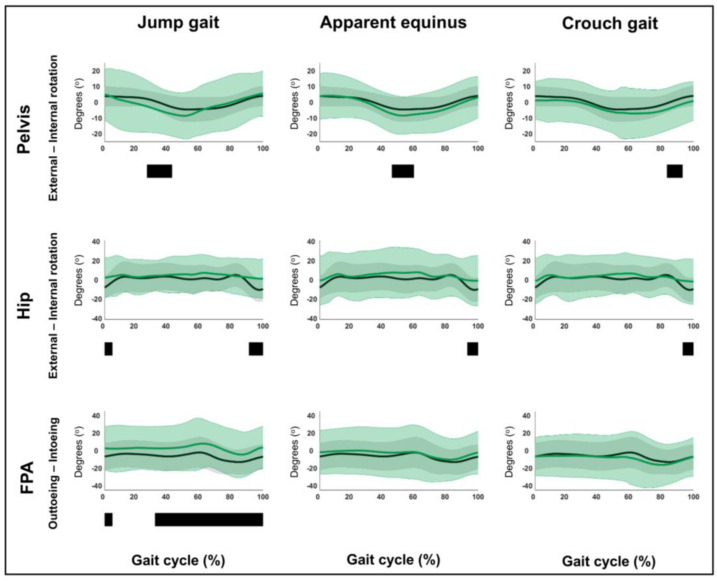
Hypothesis 1b: pattern-specific “fingerprints” are present. From left to right: transverse plane motions of the jump gait, apparent equinus, and crouch gait patterns compared to those of typically developing children. From top to bottom: the motions of the pelvis, hip, and foot progression angle (FPA) for each of these gait patterns throughout the gait cycle [*x*-axis, 0–100%; *y*-axis, degrees of motion (^o^)] are shown in green (mean ± 1 standard deviation), while the respective motions of typically developing children are shown in gray (mean ± 1 standard deviation). The results of the post hoc, statistical non-parametric comparisons (non-parametric two-sample *t*-tests) throughout the gait cycle are summarized using black bars for the statistically significant differences (i.e., suprathreshold clusters), all *p* ≤ 0.001. The details of each cluster are shown in Table S4.

The results related to hypothesis 1b are shown in Figure 4, Figure 5, Figure 6, Figure 7, Figure 8, Figure 9, Figure 10 and Figure 11 and Tables S3 and S4. Figure 4, Figure 5, Figure 6, Figure 7, Figure 8, Figure 9, Figure 10 and Figure 11 are constructed in the same way as Figure 2 and Figure 3, depicting the moments, powers or out-of-sagittal-plane motions of the seven GaP-CP gait patterns in green (mean ± 1 standard deviation), compared to those of the TD children (in gray, mean ± 1 standard deviation) throughout the gait cycle. The SnPM results (i.e., suprathreshold clusters identifying statistically significant differences between each pattern and TD gait) are summarized using black bars. The sagittal plane moment and power vectors of each pattern were statistically significantly different from TD gait. All post hoc analyses identified statistically significant differences between TD gait and all gait patterns (Figure 4, Figure 5, Figure 6 and Figure 7, *p* ≤ 0.001), apart from two (comparisons between TD gait and the mild deviations group: hip moment and knee power). In contrast to the sagittal plane kinematics, only 68% of all kinetic suprathreshold clusters identified during the stance phase exceeded the respective SEMs for more than 80% of their duration (Table S3).

The out-of-sagittal-plane kinematic differences are shown in Figure 8, Figure 9, Figure 10 and Figure 11 and Table S4. These results highlighted that in the coronal plane, deviating pelvic obliquity was found for the mild deviations and dropfoot patterns, while patients with genu recurvatum and true equinus did not show any coronal plane deviations and patients with jump gait, apparent equinus, or crouch gait displayed increased hip adduction during (pre)swing (Figure 8 and Figure 9). All patterns, apart from the mild deviations, displayed various rotational deviations from TD gait (Figure 10 and Figure 11). All comparisons exceeded the respective SEMs for over 80% of their duration.

### 3.3. Hypothesis 2a: Neighboring Gait Patterns Can Be Differentiated Based on Their Continuous Gait Waveforms

The kinematic and kinetic vectors differed among the gait patterns (non-parametric MANOVA, Table S5). The neighboring patterns showed differences only in the sagittal plane (Table S6). The most similar sagittal plane kinematic vectors were found between true equinus and jump gait, as well as between mild deviations and dropfoot. The post hoc analyses revealed that genu recurvatum and true equinus were the most similar patterns, as they differed only in the knee and ankle motion during terminal stance. Regarding kinetics, the most similar neighboring gait patterns were apparent equinus and crouch gait, where no differences were identified.

### 3.4. Hypothesis 2b: The Gait Patterns Can Be Sufficiently Differentiated from Each Other Based on Their Patient- or Impairment-Specific Characteristics

The ages at 3DGA, topographic classification, sex, and all clinical impairment scores at the time of the 3DGA differed among the gait patterns (Table 1 and Table 2). The detailed pairwise comparisons are shown in Tables S7–S11, for age, spasticity, strength, selectivity and pROM, respectively. The children with genu recurvatum, true equinus, and jump gait patterns were younger than the children with the other patterns (Table 1 and Table S7). The children with mild deviations showed the smallest impairments in most comparisons to the other gait patterns concerning spasticity, strength, and selectivity impairment scores (Tables S8–S10). Yet, regarding pROM, only 7 of 36 pairwise comparisons revealed statistically significant differences between children with mild deviations and the other patterns, three of which were found in comparison to children with crouch gait (Table S11). Interestingly, the pairwise clinical impairment comparisons were not consistently able to differentiate between the more involved patterns (genu recurvatum, true equinus, jump gait, apparent equinus, and crouch gait).

## 4. Discussion

This study examined the content and construct validity of the GaP-CP, a GCS that has been based on previously published GCSs, with the addition of a class describing “mild deviations” across all lower limb segments/joints in the sagittal plane. Hypotheses 1a and 1b were confirmed based on the analysis of the extensive study dataset, supporting the good content validity of the GaP-CP.

Overall, the comparison of the continuous kinematic waveforms of each sCP gait pattern to the gait of TD children revealed that the different patterns of the GaP-CP contained all clinically relevant gait deviations, which were in agreement with the original gait pattern definitions. Hence, the vast majority of the original classification rules could be confirmed (hypothesis 1a).

Despite the general confirmation of the original pattern definitions, detailed analyses also highlighted some discrepancies between these original definitions and the current gait data. This might stem from the fact that the original classification rules gave equal weight to all gait deviations, irrespective of whether these were primary or secondary (compensatory) deviations. For example, the primary problem of dropfoot is a lack of dorsiflexion in swing [13], while increased hip flexion in swing can be considered as a compensatory feature to facilitate foot clearance and might not always co-occur. Discrepancies between the original classification rules and observed gait deviations may also be explained by some imprecise descriptions in the original definitions, such as “hip/knee extension to a variable degree in late stance” for jump gait [10]. Such descriptions, as well as inconsistent terminology, were avoided in the refined classification rules. Henceforth, the terms “dropfoot” and “equinus” are proposed as pattern names within the GaP-CP framework, while the specific kinematic deviation, such as increased plantar flexion, should be used to describe the deviation within each pattern, including its timing within the gait cycle when relevant. An additional notable example is the decreased ankle ROM during stance, observed in the dropfoot and apparent equinus patterns. This was likely attributed to increased plantar flexion at initial contact combined with sufficient dorsiflexion at terminal stance. These consistent additional deviations necessitated slight adjustments to the original definitions. The resulting refined classification rules, summarized in Table 3, provide a comprehensive roadmap for future applications.

The need for a pattern describing mild deviations [8,9,10] was further supported by the data, as it differed from TD gait as well as from the dropfoot pattern. In contrast to previous findings [12], the limbs classified in the mild deviations pattern also exhibited pathological moments and powers (Figure 4 and Figure 6, Table S3). However, it should be noted that the mild pattern was rather heterogeneous.

Hypothesis 1b, which proposed the identification of pattern-specific “fingerprints”, was also confirmed. Apart from the gait deviations aligned with the original classification rules, additional gait deviations were identified for each pattern (Table S2). These deviations offer valuable insights and may support the classification process. However, they are not considered strict classification criteria but rather “fingerprints” of each gait pattern, as they may not directly relate to the primary issue underlying the pattern. Previous classification systems primarily focused on sagittal plane (i.e., two-dimensional) kinematic deviations [2,3], which is problematic as gait is inherently three-dimensional. While the most pronounced gait abnormalities in children with sCP often occur in the sagittal plane, it is crucial to consider deviations in the other planes as well. To address this limitation, our classification approach built upon two-dimensional gait patterns but was also the first to investigate additional fingerprints from sagittal plane kinetics and out-of-sagittal-plane kinematic deviations in these gait patterns. In this way, a more comprehensive and clinically relevant understanding of gait deviations was provided. We propose that the observed kinetic deviations should not be considered as new classification rules, as only approximately two-thirds of the identified kinetic clusters identified during stance phase exceeded the respective SEMs for more than 80% of their duration. Moreover, many of these deviations were common across multiple gait patterns. Similarly, out-of-sagittal-plane deviations, such as internal hip rotation or hip adduction, are prevalent among children with sCP [19]. Indeed, all patterns—apart from the mild deviations and dropfoot patterns—showed increased internal hip rotation, compared to TD gait, while increased hip adduction was only found in the transition from stance to swing phase for the jump gait, apparent equinus, and crouch gait patterns. The current data suggest that the majority of the out-of-sagittal-plane kinematic deviations, as well as the kinetic deviations, are not pattern-specific. Hence, they should be considered as additional features that could further complement respective gait patterns of individual patients. Whether the prevalence of deviations in the coronal and transverse planes is different for each gait pattern between children with usCP and bsCP, as previously suggested [18,45], needs further exploration. Additional explorations on the coronal, rotational, and kinetic profiles within each gait pattern are thus warranted. Moreover, also in the sagittal plane, the kinematic data revealed some more detailed features than originally described. For instance, the original definition of the crouch gait pattern described pelvic motion in stance as normal, anteriorly tilted or posteriorly tilted [10]. The current study identified a statistically significant increase in anterior pelvic tilt during mid-to-terminal stance and swing phase compared to TD gait. However, pelvic motion was highly variable, with some patients exhibiting posterior tilt, likely due to hamstring tightness. Due to this large variability, these features were defined as “fingerprints” instead of refined classification rules. Finally, future research could also explore whether different gait patterns may be combined with specific gait features not included in the GaP-CP. For example, crouch gait may be combined with altered knee motion in swing and/or altered pelvic tilt, and it remains to be defined whether such new groups would also display differences in stance phase kinematics or treatment history.

The extensive dataset was further utilized to evaluate the construct validity of the GaP-CP. Hypothesis 2a, which proposed that neighboring gait patterns could be distinguished, was confirmed. Specific differences identified between neighboring gait patterns may provide additional guidelines to enhance the classification process. Interestingly, no out-of-sagittal-plane differences were identified between the neighboring patterns, further reinforcing the conclusion that the observed kinematic deviations are not pattern-specific.

The post hoc analyses revealed that genu recurvatum and true equinus were the most similar gait patterns. Limbs exhibiting increased knee (hyper)extension during stance, with or without concurrent ankle plantar flexion, were classified as genu recurvatum [3,8,14]. This partially aligns with previous findings [12], which suggested that hyperextended knee motion is commonly accompanied by ankle motion within normal limits—a characteristic that does not align with the definition of true equinus [10].

Gait of sCP children lies along a continuum [8]. While some neighboring patterns, by definition, showed greater similarities in specific motions (e.g., knee motion), they could be differentiated based on other motions. The current study results confirmed that jump gait, apparent equinus, and crouch gait are distinct patterns [10,12], as their sagittal plane kinematic vectors were different across the entire gait cycle. Furthermore, not only did the ankle motion distinguish the patterns, but the amount of increased knee flexion during most of the stance phase also differentiated apparent equinus from crouch gait (Table S6). The increased variability in sagittal plane kinematics for jump gait is underscored not only by its original imprecise definitions (i.e., “hip/knee extension to a variable degree in late stance”) [10], but also by the observation that, although jump gait is often categorized as a “flexed” pattern [11], it resembles the true equinus pattern more closely than the apparent equinus pattern (Table S6). This suggests that jump gait represents a transitional pattern, shifting from extension toward increased flexion.

Interestingly, only a few kinetic differences were identified between the neighboring patterns. Hence, these kinetic comparisons did not allow us to further distinguish between neighboring patterns with great kinematic similarities. Similarities in kinetics point towards a similarity in muscle use, which has also been found previously for the GaP-CP patterns [36]. To clinically implement the GaP-CP, it is not only crucial to develop a classification roadmap, but also to define the clinical significance of each pattern. This can be accomplished by mapping each pattern’s clinical phenotype, which could ultimately support clinical decision-making. However, hypothesis 2b, which proposed that the gait patterns could be sufficiently distinguished based on patient- or impairment-specific characteristics, was only partially confirmed.

Genu recurvatum and true equinus were the most similar patterns in terms of age and impairment-specific characteristics, differing only in ankle dorsiflexion pROM with the knee extended (Table S11). This aligns with the sagittal plane kinematic and kinetic similarities observed between these two patterns (Table S6).

Although some patterns were originally described to characterize the gait of children with either usCP or bsCP [10,13,14], they could be found in both topographic classifications [3]. In this study, most children presenting with mild deviations, dropfoot, or true equinus showed unilateral involvement, while children with jump gait, apparent equinus, or crouch gait patterns showed bilateral involvement.

In addition, some results confirmed previous findings or reflected clinical reasoning. For instance, gait patterns showing rather minor gait deviations across the lower limb joints, such as mild deviations or dropfoot, were mostly represented by patients with higher functionality (i.e., GMFCS level I). Similarly, children with gait patterns portraying increased pathology across the hip, knee, and ankle joints, such as children walking in jump or crouch gait patterns, presented more often with lower functional capacities (GMFCS levels II-III).

Furthermore, the current study—partially—confirms previous results indicating that the clinical presentation of children with sCP deteriorates over time [10,18,19]. This age-related trend was reflected in the fact that children with crouch gait tended to be older. Bell et al. (2002) observed that children with diminished function at baseline progressed towards crouch gait over time [46]. However, it should be noted that children with mild deviations or dropfoot were also found in the oldest groups examined in this study. Longitudinal analyses are needed to establish the GaP-CP gait pattern progression over time or after treatment administration. Three recent studies have examined kinematic changes and/or clinical impairment scores modifications following treatment administration [15,16,29]. These studies demonstrated that stratifying patients based on their baseline GaP-CP gait patterns helped identify those with the greatest potential for short-term improvement. This finding has important treatment implications, suggesting that similar investigations could help determine the “best responders” for various common interventions, such as orthopedic surgery or strengthening programs. Specifically, improvements in kinematics with ankle–foot orthoses were most evident in patients with apparent equinus or crouch gait in barefoot conditions [15]. The other two studies focused on outcomes following botulinum neurotoxin type A injections and selective dorsal rhizotomy [16,29]. In the former, the most significant improvements in kinematics and clinical impairments were observed in children with jump gait and apparent equinus patterns at baseline [16], while in the latter, notable improvements were seen in children with jump gait, apparent equinus, and crouch gait at baseline [29]. However, an important question remains: how does gait classification change post-treatment? It is still unclear whether the observed kinematic improvements lead to a shift in gait pattern classification, warranting further investigation. Given that gait can change over time or in response to treatment, it may be valuable to adopt flexible or soft classification systems in the future (for example, as described in [47,48]) or to use the term “pattern recognition” rather than “pattern classification”.

While the patient-specific characteristics suggest some pattern-specific phenotypes, drawing conclusions about the impairment-specific characteristics of each gait pattern is more challenging. As expected, children with mild deviations exhibited the least impairments across most spasticity, strength, and selectivity scores compared to other gait patterns. In contrast, although the kinematic representation of true equinus was fundamentally different from patterns like apparent equinus and crouch gait, most impairment-specific scores were unable to differentiate among these patterns. Yet, it was previously shown that these patterns respond differently to treatment administration [16,29].

Establishing associations between joint contractures and gait has proven challenging [20,23,25], a difficulty also reflected in the current study’s findings. Comparisons of pROM scores revealed the fewest differences between patterns. This may be due to the more static nature of pROM measurements, which may not fully capture the dynamic aspect of gait. However, these results should be interpreted with caution, as the clinical pROM measurements were converted to an ordinal scale based on the current sample of children with sCP for the purposes of this study.

All-in-all, this study led to refined, data-driven classification rules and a comprehensive roadmap for gait pattern classification, fostering a standardized language among healthcare practitioners. For clinicians, this enhances decision-making by facilitating the selection of the most effective, patient-specific interventions, ultimately aiming to improve treatment outcomes. Caregivers and families benefit from more targeted treatment planning, minimizing unnecessary interventions and associated burdens. Most importantly, pediatric patients gain the greatest advantage, as personalized treatment strategies—such as pattern-specific orthotics or surgical planning—can enhance mobility, independence, and overall quality of life. By ensuring that children receive interventions best suited to their specific gait patterns, these findings contribute to a more effective, patient-centered approach to pediatric rehabilitation.

### Limitations

The current findings might be limited by the underrepresentation of certain gait patterns or patient-specific characteristics in the sample. For instance, the true equinus pattern was less prevalent in this study compared to previous research [10,12], and patients with GMFCS level III were also underrepresented. All the reported outcomes are specific to the sample studied and the applied methodology. For example, using average gait cycles may have introduced a smoothing effect; however, this approach was deemed most appropriate to avoid overestimating gait deviations [49]. No subgroups were created based on age or sex for either the children with sCP or the TD children. Future explorations employing larger samples could evaluate whether such subgroups would be meaningful, taking for example, the adolescence growth spurt into consideration.

Precise angle thresholds for quantitative classification rules cannot be established with SnPM and require further investigation. Future studies using larger datasets could incorporate machine learning techniques to define these thresholds, develop automatic classification algorithms, and explore soft classification approaches. The latter would be particularly valuable for patients exhibiting gait features that span neighboring patterns, such as those transitioning between different patterns. Such approaches would also allow us to investigate the stability of the classification results and could further define the robustness of the different gait patterns.

Categorizing patients based on gait patterns rather than joint-specific motion patterns may have oversimplified the complexity of gait disorders [3,6]. Conversely, detailed joint-specific deviations might represent compensatory mechanisms rather than isolated dysfunctions in need of direct intervention. Thus, it is equally essential to be able to evaluate the patient holistically. Analyzing gait patterns as a whole may simplify the classification process; it also helps to prevent an overly narrow focus on correcting a single joint, which could inadvertently diminish the functional outcome by neglecting interactions with other joints.

Moreover, longitudinal analyses, as well as the responsiveness and reliability (including inter- and intra-rater reliability with additional raters) of the GaP-CP, were beyond the scope of the present study, but are crucial next steps for the establishment of the GaP-CP as a clinically applicable tool. Another ongoing challenge is to differentiate between the primary and secondary (compensatory) causes of pathological gait patterns, which may be patient-specific rather than pattern-specific. Additionally, further exploration of the coronal, rotational, and kinetic profiles within each gait pattern is needed. Differences in other commonly assessed impairments, such as femoral anteversion, were also not included in the current analyses and should be explored in future research.

The pattern-specific phenotypes may also be influenced by various other factors, including, but not limited to, the daily use of orthoses or the frequency and duration of physical therapy, which have not been examined thus far. While some valuable insights into the clinical phenotypes associated within the GaP-CP emerged, it remains unclear whether the lower spasticity levels observed in older patients with apparent equinus or crouch gait in this study resulted from the natural evolution of sCP (i.e., gait evolving towards increased flexion [10] alongside a reduction in spasticity with age [50]), from previous spasticity-reducing treatments or a combination of both [45]. Spasticity reduction is often applied to children with sCP and has proven beneficial for several GaP-CP patterns, as it results in improvements in kinematics and spasticity without compromising the strength or selectivity of the patients with the most beneficial outcomes [16,29]. Similarly, strength training may improve gait, particularly for patients with jump gait, followed by those with genu recurvatum and crouch gait, who exhibited the lowest composite strength scores. The analyses conducted in this study do not allow for the exploration of relationships between patient- or impairment-specific characteristics and the GaP-CP gait patterns. Future studies should employ advanced analyses to examine these relationships, which could help clarify potential causal links between the clinical impairments and the pattern-specific gait deviations. Lastly, future studies involving samples from other research centers could help assess the generalizability of the current findings and establish the GaP-CP as a universally applicable tool. Investigating the pattern-specific phenotypes in samples from different research centers will be a crucial next step in understanding how treatment history and patient- and impairment-specific characteristics influence the gait patterns of children with sCP.

## 5. Conclusions

This study established both the content and construct validity of the GaP-CP, a clinical GCS for children with sCP. The study led to refined, data-driven classification rules, along with additional, frequently observed gait deviations (i.e., fingerprints), which form a comprehensive roadmap for classification. By standardizing pattern-specific classification rules, a common language among healthcare practitioners is established, facilitating its potential integration into the clinical decision-making process. Key kinematic and kinetic differences between neighboring gait patterns were clearly identified, along with variations in patient- or impairment-specific characteristics, marking an initial step toward developing pattern-specific clinical phenotypes. These phenotypes provide further evidence of the Gap-CP construct validity and, along with the classification roadmap, they offer a foundation for implementing this GCS as a valuable tool in the clinical decision-making regarding children with sCP.

## Figures and Tables

**Table 1:** General characteristics for typically developing children (N = 56) and children with spastic cerebral palsy (N = 270).

			TD Sample	Total Sample	Mild Deviations	Dropfoot	Genu Recurvatum	True Equinus	Jump Gait	Apparent Equinus	Crouch Gait	*p*-Value (KW) ^a^	*p*-Value (χ^2^) ^a^
	**Kinematics**	[N (%)]	56 (100%)	270 (100%)	33 (12.2%)	28 (10.4%)	51 (18.9%)	17 (6.3%)	28 (10.4%)	71 (26.3%)	42 (15.5%)		
	**Kinetics**	[N (%)]	56 (100%)	208 (100%)	31 (14.9%)	27 (13%)	39 (18.8%)	15 (7.2%)	15 (7.2%)	57 (27.4%)	24 (11.5%)		
**General characteristics**	**Age at 3DGA**	Median (IQR)	10.7 (7.8–14.7)	7.7 (5.9–10.8)	9.2 (7.4–11.5)	8.7 (6.4–11.0)	6.3 (5.2–7.7)	6.3 (4.9–9.3)	6.2 (5.2–7.7)	8.6 (6.4–11.7)	10.4 (7.5–12.5)	**<0.001**	
	**Topographic classification**												**<0.001**
	**usCP**	[N (%)]	NA	123 (45.5%)	21 (63.6%)	23 (82.1%)	24 (47.1%)	10 (58.8%)	3 (10.7%)	27 (38.0%)	15 (35.7%)		
	**bsCP**	[N (%)]		147 (54.5%)	12 (36.4%)	5 (17.9%)	27 (52.9%)	7 (41.2%)	25 (89.3%)	44 (62.0%)	27 (64.3%)		
	**Sex**												0.403
	**Boys**	[N (%)]	24 (42.9%)	160 (59.3%)	20 (60.6%)	17 (60.7%)	25 (49.0%)	9 (52.9%)	20 (71.4%)	40 (56.3%)	29 (69.0%)		
	**Girls**	[N (%)]	32 (57.1%)	110 (40.7%)	13 (39.4%)	11 (39.3%)	26 (51.0%)	8 (47.1%)	8 (28.6%)	31 (43.7%)	13 (31.0%)		
	**GMFCS**												**<0.001**
	**I**	[N (%)]	NA	157 (58.1%)	32 (97.0%)	25 (89.3%)	26 (51.0%)	7 (41.2%)	7 (25.0%)	43 (60.6%)	17 (40.5%)		
	**II**	[N (%)]		82 (30.4%)	1 (3.0%)	3 (10.7%)	20 (39.2%)	9 (52.9%)	13 (46.4%)	22 (31.0%)	14 (33.3%)		
	**III ^b^**	[N (%)]		31 (11.5%)	0 (0.0%)	0 (0.0%)	5 (9.8%)	1 (5.9%)	8 (28.6%)	6 (8.5%)	11 (26.2%)		
**Gait trials’ information**	**Kinematics**												
	**Gait cycles used ^c^**	Median (IQR)	3 (2–3)	4 (3–5)	5 (3–5)	4 (3–6)	4 (3–5)	5 (3–6)	3 (3–4)	4 (3–6)	4 (3–5)	0.374	
	**Side used ^c^**											0.848	
	**Left**	[N (%)]	26 (46.4%)	124 (45.9%)	15 (12.1%)	13 (10.5%)	28 (22.6%)	8 (6.4%)	11 (8.9%)	32 (25.8%)	17 (13.7%)		
	**Right**	[N (%)]	30 (53.6%)	146 (54.1%)	18 (12.3%)	15 (10.3%)	23 (15.8%)	9 (6.2%)	17 (11.6%)	39 (26.7%)	25 (17.1%)		
	**Kinetics**												
	**Gait cycles used ^c^**	Median (IQR)	3 (2–3)	2 (2–3)	3 (2–3)	2 (1–3)	2 (1–3)	2 (2–3)	2 (2–3)	2 (1–3)	3 (2–3)	0.553	
	**Side used ^c^**											0.600	
	**Left**	[N (%)]	26 (46.4%)	98 (47.1%)	14 (45.2%)	12 (44.4%)	23 (59%)	8 (53.3%)	5 (33.3%)	27 (47.4%)	9 (37.5%)		
	**Right**	[N (%)]	30 (53.6%)	110 (52.9%)	17 (54.8%)	15 (55.6%)	16 (41%)	7 (46.7%)	10 (66.7%)	30 (52.6%)	15 (62.5%)		

The nominal characteristics are presented as the number of observations (N) and respective percentages (%). The continuous characteristics are presented as medians and their respective interquartile ranges (IQRs). Kruskal–Wallis (KW) comparisons were used for continuous variables; and chi-squared (χ^2^) tests were used for nominal or ordinal variables. ^a^ All comparisons were carried out among the different gait pattern groups. The statistically significant *p*-values are indicated in bold font. ^b^ All patients with GMFCS level III used a kayewalker as an assistive device during the 3DGA. ^c^ Gait cycles or sides used to create the averaged gait cycle. KW: Kruskal–Wallis test; χ^2^: chi-squared test; 3DGA: three-dimensional gait analysis; IQR: interquartile range; usCP: unilateral spastic cerebral palsy; bsCP: bilateral spastic cerebral palsy; GMFCS: gross motor function classification system.

**Table 2:** Detailed clinical impairment scores for children with spastic cerebral palsy (N = 270).

		Total Sample	Mild Deviations	Dropfoot	Genu Recurvatum	True Equinus	Jump Gait	Apparent Equinus	Crouch Gait	*p*-Value (KW)
**Spasticity**	**Composite spasticity**	4.5 (3.5–6)	3 (2.5–3.8)	3.5 (3–4.5)	4.5 (3.5–6.5)	4.5 (3.5–7)	6.5 (5.5–7.5)	4.5 (3.5–6)	4.5 (3.5–7)	<0.001
	**Psoas**	1 (0–1.5)	0 (0–1)	0 (0–1)	1 (0–1)	1 (0–1.5)	1.5 (1–1.5)	1 (0–1.5)	1 (0–1.5)	<0.001
	**Hamstrings**	1.5 (1–2)	1.5 (1–1.5)	1 (1–1.5)	1.5 (1–2)	1.5 (1–2)	2 (1.5–2)	1.5 (1–2)	1.5 (1–2)	<0.001
	**Rectus femoris**	0 (0–1)	0 (0–0)	0 (0–0)	0 (0–1.5)	0 (0–1)	1 (0–1.5)	1 (0–1)	1 (0–1.5)	<0.001
	**Gastrocnemius**	1.5 (1.5–2)	1.5 (1.5–1.5)	1.5 (1.5–2)	1.5 (1.5–2)	2 (1.5–3)	2 (2–3)	1.5 (1.5–2)	1.5 (1.5–2)	<0.001
	**Soleus**	1.5 (1.5–2)	1.5 (1–1.5)	1.5 (1.5–2)	1.5 (1.5–2)	2 (1.5–2)	2 (1.5–3)	1.5 (1.5–1.5)	1.5 (1–1.5)	<0.001
**Weakness**	**Composite weakness**	22 (19–23)	25 (23–26)	23 (22–24)	20 (19–22)	20 (17–21)	18 (17–20)	22 (19–23)	21 (18–22)	<0.001
	**Hip flexors**	4 (4–4)	4 (4–5)	4 (4–5)	4 (3–4)	4 (3–4)	4 (3–4)	4 (4–4)	4 (3–4)	<0.001
	**Hip extensors**	4 (3–4)	4 (4–5)	4 (4–4)	3 (3–4)	3 (3–4)	3 (3–4)	4 (3–4)	3 (3–4)	<0.001
	**Knee flexors**	4 (3–4)	4 (4–5)	4 (4–4)	4 (3–4)	3 (3–4)	3 (3–3)	4 (3–4)	3 (3–4)	<0.001
	**Knee extensors**	4 (3–4)	4 (4–5)	4 (4–4)	4 (4–4)	4 (3–4)	3 (3–4)	4 (3–4)	4 (3–4)	<0.001
	**Ankle DF90**	3 (3–4)	4 (4–4)	3 (3–4)	3 (2–3)	3 (2–3)	3 (2–3)	3 (3–4)	3 (3–4)	<0.001
	**Ankle DF0**	3 (3–4)	4 (4–4)	3 (3–4)	3 (2–3)	3 (2–3)	3 (2–3)	3 (3–4)	3 (3–3)	<0.001
	**Ankle PF**	3 (3–4)	4 (3–4)	4 (3–4)	3 (3–3)	3 (3–3)	3 (2–3)	3 (3–4)	3 (3–3)	<0.001
**Selectivity**	**Composite selectivity**	10 (8–11)	11.5 (11–12)	10.5 (10–11)	9 (8–10.5)	9.5 (7.5–10)	8 (6.5–9)	10.5 (8–11)	9 (8–10.5)	<0.001
	**Hip flexors**	2 (1.5–2)	2 (2–2)	2 (2–2)	2 (1.5–2)	2 (1.5–2)	2 (1.5–2)	2 (2–2)	2 (1.5–2)	<0.001
	**Hip extensors**	2 (1.5–2)	2 (2–2)	2 (2–2)	1.5 (1.5–2)	2 (1.5–2)	1.5 (1–2)	2 (1.5–2)	2 (1–2)	<0.001
	**Knee flexors**	2 (1.5–2)	2 (2–2)	2 (2–2)	1.5 (1.5–2)	1.5 (1.5–2)	1.5 (1.5–1.5)	2 (1.5–2)	1.5 (1–2)	<0.001
	**Knee extensors**	2 (1.5–2)	2 (2–2)	2 (2–2)	2 (1.5–2)	1.5 (1.5–2)	1.5 (1–1.5)	2 (1.5–2)	1.5 (1.5–2)	<0.001
	**Ankle DF90**	1.5 (1–1.5)	2 (1.5–2)	1.5 (1–1.5)	1 (1–1.5)	1 (0.5–1.5)	1 (0.5–1)	1.5 (1–1.5)	1.5 (1–1.5)	<0.001
	**Ankle DF0**	1.5 (1–1.5)	2 (1.5–2)	1.5 (1–1.5)	1 (0.5–1.5)	1 (0.5–1.5)	1 (0.5–1)	1.5 (1–1.5)	1.5 (1–1.5)	<0.001
	**Ankle PF**	1.5 (1–2)	2 (1.5–2)	1.5 (1.5–2)	1 (1–1.5)	1.5 (1–1.5)	1 (1–1.5)	1.5 (1–2)	1.5 (1–1.5)	<0.001
**pROM**	**Composite pROM**	3 (2–4)	2 (2–3)	3 (2–3)	2 (1–3)	3 (2–3)	3 (2–4)	3 (2–4)	3 (2–4)	0.029
	**Hip extensors**	0 (0–1)	0 (0–0)	0 (0–0)	0 (0–1)	0 (0–1)	0 (0–2)	0 (0–1)	0 (0–2)	0.004
	**Upopl**	1 (1–2)	1 (0–1)	1 (0–1)	1 (0–1)	1 (0–1)	1 (1–2)	1 (1–2)	1 (1–2)	<0.001
	**Bpopl**	1 (1–2)	1 (0–2)	1 (0–1)	1 (0–1)	1 (0–1)	1 (1–2)	1 (1–2)	2 (1–2)	0.001
	**Ankle DF90**	1 (1–2)	1 (1–2)	2 (1–2)	1 (1–2)	1 (1–2)	1 (0–2)	1 (1–2)	1 (0–1)	<0.001
	**Ankle DF0**	1 (1–2)	1 (1–1)	1 (1–2)	1 (1–1)	2 (1–2)	1 (1–2)	1 (1–2)	1 (0–1)	0.005

Composite impairment scores were constructed according to [22] for children with cerebral palsy. Typically developing children do not display impairments. Medians and interquartile ranges are shown for all impairment scores. All comparisons were carried out among the different gait pattern groups, all *p* ≤ 0.05. KW: Kruskal–Wallis; DF90: ankle dorsiflexors with the knee in 90° flexion; DF0: ankle dorsiflexors with the knee extended; PF: plantar flexors; pROM: passive range of motion; Upopl: unilateral popliteal angle; Bpopl: bilateral popliteal angle.

**Table 3:** Original classification rules, identified kinematic deviations from typically developing gait and final set of refined classification rules.

Gait Pattern	Segment or Joint	Original Classification Rules	Kinematic Deviations		Notes	Refined Classification Rules
		Deviation	Deviation	Phase (%)		
**Mild ** **deviations**	**Pelvis**	No or minor deviations throughout GC	Increased anterior tilt	ISw–MSw (16.9%)		No or minor deviations throughout GC; possible increased anterior tilt in ISw–MSw
	**Hip**	No or minor deviations throughout GC	Decreased Ext in St	TSt (20.8%)		No or minor deviations throughout GC; possible decreased Ext in TSt
	**Knee**	No or minor deviations throughout GC	Increased FL	IC–LR (5.2%)		No or minor deviations throughout GC; possible increased FL in IC–LR and TSw
				TSw (16.9%)		
	**Ankle**	No or minor deviations throughout GC	Increased DFL	PSw–MSw (21.5%)		No or minor deviations throughout GC; possible increased DFL in PSw–MSw
**Dropfoot**	**Pelvis**	Increased lordosis throughout GC	Increased anterior tilt	Entire GC (96.9%)		Increased anterior tilt throughout GC
	**Hip**	Increased FL in Sw	Increased FL in Sw	ISw–TSw (33.3%)		Increased FL in Sw
	**Knee**	Increased FL at TSw, IC and LR	Increased FL at TSw, IC and LR	IC–LR (10.2%)		Increased FL at TSw, IC and LR
				MSw–TSw (22.9%)		
	**Ankle**	Increased PFL in Sw	Increased PFL in Sw	MSw–TSw (24.8%)		Increased PFL in Sw; normal or reduced ROM during St
		Adequate DFL ROM in St	Reduced ROM in St	N/A	Discrete parameter, analysis N/A with SnPM	
**Genu ** **recurvatum**	**Pelvis**	N/A	N/A	-		No or minor deviations throughout GC, possible increased anterior tilt
	**Hip**	Almost normal motion in St	Almost normal motion in St	-	No deviations from TD	No or minor deviations in St
	**Knee**	Full knee Ext or HE in St	Full knee Ext or HE in St	MSt–PSw (44%)	Cluster expands to Sw [MSt–MSw (59%)]—Table S2	Full knee Ext or HE in St
	**Ankle**	Impaired motor control, PFL or reduced DFL	Decreased DFL	MSt–PSw (27.8%)		Decreased DFL in MSt-TSt
**True ** **equinus**	**Pelvis**	Normal ROM or anterior tilt	Increased anterior tilt	LR–PSw (53.9%)	Normal ROM is also possible; cluster expands to Sw [LR–TSw (83.1%)]—Table S2	Normal ROM or increased anterior tilt
	**Hip**	Full hip Ext	Decreased Ext in TSt	TSt (7.8%)		Decreased hip Ext in TSt
	**Knee**	Full knee Ext	Full knee Ext	-		Full knee Ext
	**Ankle**	Equinus during St	Increased PFL in St	MSt–PSw (39.7%)		Increased PFL in St
**Jump ** **gait**	**Pelvis**	Normal ROM or anterior tilt	Increased anterior tilt	LR–PSw (57.9%)	Normal ROM is also possible; cluster expands to Sw [LR–TSw (88.2%)]—Table S2	Normal ROM or increased anterior tilt
	**Hip**	Increased FL in early St	Increased FL in early St	IC–LR (8%)		Increased FL in St
		Followed by Ext to a variable degree	Decreased Ext in MSt–PSw	MSt–PSw (39.1%)	Cluster expands to Sw [MSt–TSw (79.1%)]—Table S2	
	**Knee**	Increased FL in early St	Increased FL in St	IC–LR (10%)		Increased FL in St
		Followed by Ext to a variable degree	Decreased Ext in MSt–PSw	MSt–PSw (56.9%)		
	**Ankle**	Equinus, particularly in late St	Increased PFL in late St	MSt–PSw (37.1%)		Increased PFL in late St
**Apparent ** **equinus**	**Pelvis**	Normal ROM or anterior tilt	Increased anterior tilt	St (60%)	Normal ROM is also possible; cluster expands to Sw [GC (100%)]—Table S2	Normal ROM or increased anterior tilt
	**Hip**	Increased FL in St	Increased FL in St	St (60%)	Cluster expands to Sw [GC (100%)]—Table S2	Increased FL in St
	**Knee**	Increased FL in St	Increased FL in St	St (60%)	Cluster expands to Sw [IC–ISw (60.6%)]—Table S2	Increased FL in St
	**Ankle**	Normal ROM in St	Reduced ROM in St	N/A	Discrete parameter, analysis N/A with SnPM	Normal or reduced ROM during St
**Crouch ** **gait**	**Pelvis**	Normal ROM, anterior or posterior tilt	Increased anterior tilt	MSt–TSt (28.1%)		Normal ROM, anterior or posterior tilt
	**Hip**	Increased FL in St	Increased FL in St	St (60%)	Cluster expands to Sw [GC (100%)]—Table S2	Increased FL in St
	**Knee**	Increased FL in St	Increased FL in St	St (60%)	Cluster expands to Sw [IC–ISw (64.6%)]—Table S2	Increased FL in St
	**Ankle**	Excessive DFL in St	Increased DFL in St	St (60%)	Cluster expands to Sw [IC–TSw (93.7%)]—Table S2	Increased DFL in St

The kinematic deviations that confirmed or contradicted the original classification rules [3] are indicated in bold or italics, respectively. Typically developing gait was used as a reference regarding the observed gait deviations. For example, the hip in typically developing children is extended in TSt; if this was not achieved for a certain pattern, this was labeled as “Decreased Ext”. Abbreviations: GC: gait cycle; ISw: initial swing; MSw: midswing; Ext: extension; St: stance; TSt: terminal stance; FL: flexion; IC: initial contact; LR: loading response; TSw: terminal swing; DFL: dorsiflexion; PSw: preswing; MSt: midstance; Sw: swing; ROM: range of motion; N/A: non-applicable; SnPM: statistical non-parametric mapping; HE: hyperextension; PFL: plantar flexion.

## Data Availability

The data presented in this study are available on request from the corresponding author. The data are not publicly available due to restrictions, e.g., privacy or ethical.

## Supplementary Materials

The following supporting information can be downloaded at: https://www.mdpi.com/article/10.3390/children12030269/s1, Table S1. Original classification rules and description of the classification procedure; Table S2. Hypothesis 1a: Content validity—sagittal plane kinematic deviations between typically developing gait (N = 56) and children with spastic cerebral palsy (N = 270); Table S3. Hypothesis 1b: Content validity—sagittal plane kinetic deviations between typically developing gait (N = 56) and children with spastic cerebral palsy (N = 208); Table S4. Hypothesis 1b: Content validity—coronal and transverse plane kinematic deviations between typically developing gait (N = 56) and children with spastic cerebral palsy (N = 270); Table S5. Hypothesis 2a: Construct validity —kinematic (N = 270) and kinetic (N = 208) vector comparisons among all spastic cerebral palsy gait patterns; Table S6. Hypothesis 2a: Construct validity—sagittal plane kinematic (N = 270) and kinetic (N = 208) deviations: comparisons between neighboring gait patterns; Table S7. Hypothesis 2b: Differences in age—pairwise comparisons between gait patterns; Table S8. Hypothesis 2b: Differences in spasticity impairment scores—pairwise comparisons between gait patterns; Table S9. Hypothesis 2b: Differences in strength impairment scores—pairwise comparisons between gait patterns; Table S10. Hypothesis 2b: Differences in selectivity impairment scores—pairwise comparisons between gait patterns; Table S11. Hypothesis 2b: Differences in pROM impairment scores—pairwise comparisons between gait patterns. References [3,43,44,51] are cited in the Supplementary Materials.
