# Supplementary material for: The Gait Pattern Classification System for Children with Spastic Cerebral Palsy (GaP-CP)—A Validity Study
Source: Children (Basel). 2025 Feb 23;12(3):269. doi: 10.3390/children12030269 (PMC11941407; doi:10.3390/children12030269)
Supplement: Supplementary file 1 [file children-12-00269-s001.zip › children-3392115-supplementary.pdf]

Table S1

Original classification rules[a] and description of the classification procedure

Gait pattern	Joint	Original classification rules[a]	Classification procedure
**Mild deviations**	Pelvis	No or minor deviations throughout GC	No or minor deviations throughout GC, i.e., deviations not exceeding the TD ±1 SD bands **OR** only one gait deviation slightly exceeding the TD ±1 SD bands (e.g., increased knee FL at IC).
	Hip	No or minor deviations throughout GC	
	Knee	No or minor deviations throughout GC	
	Ankle	No or minor deviations throughout GC	
**Dropfoot[a]**	Pelvis	Increased lordosis throughout GC	Increased PFL in Sw, with adequate DFL ROM in St, accompanied by: i) increased pelvic anterior tilt (above the TD +1 SD or on the higher end of the SD band), ii) increased hip FL in Sw and knee FL in TSw, IC and LR (or on the higher end of the SD band).
	Hip	Increased FL in Sw	
	Knee	Increased FL at TSw, IC and LR	
	Ankle	Increased PFL in Sw<br>Adequate DFL ROM in St	
**Genu recurvatum[a]**	Pelvis	N/A	Full knee Ext or HE combined with ankle PFL or reduced DFL. Pelvic and hip motions were not considered.
	Hip	Almost normal motion in St	
	Knee	Full knee Ext or HE in St	
	Ankle	Impaired motor control, PFL or reduced DFL	
**True equinus[a]**	Pelvis	Normal ROM or anterior tilt	Ankle PFL during St, accompanied by: i) knee Ext (knee motion within the TD ±1 SD bands and **NOT** in HE), ii) hip motion towards Ext, and iii) pelvis in normal or anterior tilt.
	Hip	Full hip Ext	
	Knee	Full knee Ext	
	Ankle	Equinus during St	
**Jump gait[a]**	Pelvis	Normal ROM or anterior tilt	Ankle PFL, particularly in late St, accompanied by: i) early hip and knee FL, followed by hip or knee motion towards Ext, but not necessarily reaching the TD ±1 SD bands, and ii) pelvis in normal or anterior tilt.
	Hip	Increased FL in early St<br>Followed by Ext to a variable degree	
	Knee	Increased FL in early St<br>Followed by Ext to a variable degree	
	Ankle	Equinus, particularly in late St	
**Apparent equinus[a]**	Pelvis	Normal ROM or anterior tilt	No ankle deviations towards increased DFL or PFL (e.g., horizontal second ankle rocker, or ankle motion following that of the TD sample), accompanied by: i) increased hip and knee FL in St, and ii) pelvis in normal or anterior tilt.
	Hip	Increased FL in St	
	Knee	Increased FL in St	
	Ankle	Normal ROM in St	
**Crouch gait[a]**	Pelvis	Normal ROM, anterior or posterior tilt	Ankle in excessive DFL or at the highest end of the TD +1 SD, accompanied by increased knee and hip FL in St **OR** by decreased hip ROM. Pelvic motion was not considered as a classification criterion
	Hip	Increased FL in St	
	Knee	Increased FL in St	
	Ankle	Excessive DFL	

Typically developing gait was used as a reference regarding the observed gait deviations. For example, the hip in typically developing children is extended in terminal St; if this was not achieved for a certain pattern, this was labelled as "Decreased Ext".

[a]Original classification rules stem from [1].

Abbreviations: GC: gait cycle; TD: typically developing; SD: standard deviation; FL: flexion; IC: initial contact; Sw: swing; TSw: terminal swing; LR: loading response; PFL: plantar flexion; DFL: dorsiflexion; ROM: range of motion; N/A: non-applicable; St: stance; Ext: extension; HE: hyperextension.

Table S2

Hypothesis 1a: Content validity - Sagittal plane kinematic deviations between typically developing gait (N=56) and children with spastic cerebral palsy (N=270).

			Mild deviations (n=33)	Dropfoot (n=28)	Genu recurvatum (n=51)	True equinus (n=17)	Jump gait (n=28)	Apparent equinus (n=71)	Crouch gait (n=42)
Statistical non-parametric mapping	**Vector[a]**	**n clusters (T2*)**	2 (22.5)	2 (24.3)	1 (21.2)	1 (29.7)	1 (23.2)	1 (21.4)	1 (21.1)
		**% extent (range)**	10 (0 - 10)	50.6 (0 - 50.6)	100 (0 - 100)	100 (0 -100)	100 (0 - 100)	100 (0 - 100)	100 (0 - 100)
			67.4 (32.6 - 100)	46.3 (53.7 - 100)					
		**Total % extent**	77.4	96.9	100	100	100	100	100
	**Pelvis[b]**	**n clusters (t*)**	1 (3.5)	1 (3.7)	1 (3.76)	1 (3.6)	1 (3.4)	1 (4.0)	2 (3.3)
		**% extent (range)**	16.9 (60.3 - 77.2)	63.4 (17.3 - 80.7)	100 (0 - 100)	83.1 (6.1 - 89.2)	88.2 (2.1 - 90.3)	100 (0 - 100)	28.1 (9.9 - 38)
									30.5 (53.6 - 84.1)
		**Total % extent**	16.9	63.4	100	83.1	88.2	100	58.6
	**Hip[b]**	**n clusters (t*)**	1 (3.8)	2 (3.7)	1 (3.74)	2 (3.9)	2 (3.5)	1 (4.2)	1 (3.5)
		**% extent (range)**	20.8 (31 - 51.8)	22.1 (29.5 - 51.6)	22.4 (75.3 - 97.7)	7.8 (38.6 - 46.4)	8 (0 - 8)	100 (0 - 100)	100 (0 - 100)
				33.3 (66.7 - 100)		17.6 (80 - 97.6)	79.1 (20.9 - 100)		
		**Total % extent**	20.8	55.4	22.4	25.4	87.1	100	100
	**Knee[b]**	**n clusters (t*)**	2 (4.0)	2 (4.0)	3 (3.9)	3 (4.0)	3 (3.9)	2 (3.8)	2 (3.7)
		**% extent (range)**	5.2 (0 - 5.2)	10.2 (0 - 10.2)	4.2 (0 - 4.2)	5.8 (0 - 5.8)	56.9 (0 - 56.9)	60.6 (0 - 60.6)	64.6 (0 - 64.6)
			16.9 (83.1 - 100)	22.9 (77.1 - 100)	59 (16 - 75)	3.5 (69.3 - 72.8)	8.5 (66.9 - 75.4)	20.1 (79.9 - 100)	20.4 (79.6 - 100)
					18.5 (81.5 - 100)	16.2 (83.8 - 100)	19.9 (80.1 - 100)		
		**Total % extent**	22.1	33.1	81.7	25.5	85.3	80.7	85
	**Ankle[b]**	**n clusters (t*)**	1 (4.1)	1 (4.0)	2 (3.9)	2 (4.8)	3 (3.0)	3 (4.0)	1 (3.9)
		**% extent (range)**	21.5 (52 - 73.5)	24.8 (75.2 - 100)	27.8 (22.9 - 50.7)	39.7 (18.4 - 58.1)	3 (6.2 - 9.2)	20 (2.2 - 22.2)	93.7 (0 - 93.7)
					25.3 (74.7 - 100)	30 (70 - 100)	37.1 (22.9 - 60)	13.9 (55.2 - 69.1)	
							31.8 (68.2 - 100)	4.5 (93.3 - 97.8)	
		**Total % extent**	21.5	24.8	53.1	69.7	71.9	38.4	93.7

			TD (n=56)	Dropfoot (n-28)	Apparent equinus (n=71)
MWU	**aROMSt[c]**	**Median (IQR)**	24.2 (21.2 - 27.1)	19.9 (17.0 - 22.6)	18.9 (15.0 - 21.1)

$^{a}\alpha = 0.007$; $^{b}\alpha = 0.002$; $^{c}\alpha = 0.05$.

all $p \leq 0.001$.

All statistically significant clusters of segments or joints were additionally judged based on whether they identified differences larger than the standard errors of measurement (SEM) for the entire gait cycle, suggested by Everaert et al. (2024) [2]. All identified clusters exceeded the respective SEM for more than 80% of their duration. Clusters highlighted in blue indicate pattern-specific "fingerprints" (i.e., additional prevalent kinematic deviations from TD gait).

Vector: component consisting of the combination of the individual sagittal plane motions of the pelvis, hip, knee, and ankle; n clusters: number of identified suprathreshold clusters; T2*/t*: critical thresholds needed to reject the null hypothesis; % extent (range): extent of the identified suprathreshold cluster (start and end points of the identified cluster); MWU: Mann-Whitney U test; TD: typically developing children; aROMSt: ankle range of motion during stance; IQR: interquartile range.

Table S3

Hypothesis 1b: Content validity - Sagittal plane kinetic deviations between typically developing gait (N=56) and children with spastic cerebral palsy (N=208).

			Mild deviations (n=31)	Dropfoot (n=27)	Genu recurvatum (n=39)	True equinus (n=15)	Jump gait (n=15)	Apparent equinus (n=57)	Crouch gait (n=24)
**Moments**	**Vector**[a]	**n clusters (T2*)**	3 (23.2)	3 (23.5)	2 (22.1)	4 (33.1)	4 (31.6)	2 (21.2)	3 (24.6)
		**% extent (range)**	14.6 (33.9 - 48.5)	49.6 (0.2 - 49.8)	57.9 (0 - 57.9)	55.5 (0.3 - 55.8)	25.5 (0.1 - 25.6)	63.5 (0.2 - 63.7)	53.4 (0.2 - 53.6)
			5.1 (58.6 - 63.7)	26 (67 - 93)	40 (60 - 100)	3.2 (61.1 - 64.3)	30.3 (27.3 - 57.6)	36 (64 - 100)	30.5 (60.7 - 91.2)
			13.4 (64.8 - 78.2)	3.8 (96.2 - 100)		27.4 (67.6 - 95)	5.7 (59.2 - 65)		5.4 (94.6 - 100)
						3.7 (96.3 - 100)	30.2 (65 - 95.2)		
		**Total % extent**	33.1	79.4	97.9	89.8	91.7	99.5	89.3
	**Hip**[b]	**n clusters (t*)**	-	1 (4.4)	3 (4.2)	1 (4.7)	3 (4.9)	2 (4.2)	2 (4.4)
		**% extent (range)**		25.6 (22.7 - 48.3)	3.3 (0.2 - 3.55)	32.6 (19.4 - 52)	4.9 (25.7 - 30.6)	32.7 (20.2 - 52.9)	25.1 (25.8 - 50.9)
					22.5 (27.7 - 50.2)		3.2 (65 - 68.2)	7.4 (73.2 - 80.6)	8 (72.5 - 80.5)
					8.4 (60.7 - 69.1)		5.5 (73.8 - 79.3)		
		**Total % extent**			34.2	32.6	13.6	40.1	
	**Knee**[b]	**n clusters (t*)**	1 (4.2)	2 (4.2)	5 (3.63)	3 (4.6)	3 (4.8)	4 (4.1)	3 (4.4)
		**% extent (range)**	5.6 (68.6 - 74.2)	9.6 (17.7 - 27.3)	3.5 (0 - 3.5)	17.6 (12.2 - 29.8)	24.8 (33.5 - 58.3)	3.4 (0.7 - 4.1)	4.2 (0.5 - 4.7)
				15.3 (62.1 - 77.4)	25.9 (10.6 - 36.5)	13.3 (66.3 - 79.6)	12.2 (65.1 - 77.3)	18.3 (32.8 - 51.1)	23.5 (26.5 - 50)
					4.9 (48.8 - 53.7)	6.9 (88 - 94.9)	7.3 (86.2 - 93.4)	3.2 (54.6 - 57.8)	13 (64.8 - 77.8)
					15.2 (64.2 - 79.4)			14 (65.4 - 79.4)	
		**Total % extent**	5.6	24.9	14.2 (85.8 - 100)	37.8	44.3	38.9	40.7
	**Ankle**[b]	**n clusters (t*)**	3 (4.2)	4 (4.4)	5 (4.1)	4 (4.7)	5 (4.9)	5 (4.2)	5 (4.6)
		**% extent (range)**	11.1 (37.8 - 48.9)	23.7 (0.5 - 24.2)	23.3 (0.7 - 24)	22.2 (0.6 - 22.8)	23.2 (0.1 - 23.3)	27.3 (0.8 - 28.1)	22.3 (0.8 - 23.1)
			5 (58.2 - 63.2)	12.7 (37.2 - 49.9)	20 (35.4 - 55.4)	19.9 (36.6 - 56.5)	21.6 (35.1 - 56.7)	17.5 (36.9 - 54.4)	9 (41.4 - 50.4)
			6.1 (64.7 - 70.8)	10.8 (81.8 - 92.6)	7.7 (60.3 - 68)	13.7 (78.5 - 92.2)	3.6 (61.6 - 65.2)	9.4 (63.5 - 72.9)	8.2 (61.1 - 69.3)
				3.5 (96.5 - 100)	15.2 (77.8 - 93)	3.4 (96.6 - 100)	6.3 (86.9 - 75.2)	12.8 (79.9 - 92.6)	8.2 (82.1 - 90.3)
					4 (96 - 100)		12.4 (79.8 - 92.2)	5 (95 - 100)	5 (95 - 100)
		**Total % extent**	22.2	50.7	70.2	59.2	67.1	72	52.7

Table S3 ***(continued)***

			Mild deviations (n=31)	Dropfoot (n=27)	Genu recurvatum (n=39)	True equinus (n=15)	Jump gait (n=15)	Apparent equinus (n=57)	Crouch gait (n=24)
**Powers**	**Vector**[a]	**n clusters (T2*)**	2 (23.9)	5 (25.2)	7 (22.4)	5 (32.0)	6 (32.7)	5 (21.4)	6 (25.1)
		**% extent (range)**	10 (27.9 - 37.9)<br>11.6 (44.6 - 56.2)<br>12.1 (67.5 - 79.6)	10.8 (4.5 - 15.3)<br>19.2 (19.1 - 38.3)<br>11.2 (44.5 - 55.7)<br>7 (74.1 - 81.1)<br>3.6 (93 - 96.6)	11.8 (0 - 11.8)<br>23.2 (17.5 - 40.7)<br>13.4 (45.4 - 58.8)<br>3.1 (60.6 - 63.7)<br>6.2 (64.4 - 70.6)<br>7.1 (73.4 - 80.5)<br>15 (85 - 100)	6.4 (4.2 - 10.6)<br>23.2 (15.6 - 38.8)<br>9.4 (48.1 - 57.5)<br>4.8 (74.1 - 78.9)<br>13.9 (86.1 - 100)	12.1 (0 - 12.1)<br>42.5 (15.2 - 57.7)<br>9.4 (61.7 - 71.1)<br>6.7 (73.2 - 79.9)<br>9.2 (85.7 - 94.9)<br>3.1 (96.9 - 100)	13.9 (0 - 13.9)<br>23.1 (19 - 42.1)<br>13.8 (44.2 - 58)<br>20.6 (60.1 - 81.7)<br>11.7 (84.9 - 96.6)	3.5 (0 - 3.5)<br>9.1 (5.7 - 14.8)<br>14.9 (24.1 - 39)<br>12.3 (44.6 - 56.9)<br>19.6 (61.2 - 80.8)<br>7.3 (87.3 - 94.6)
		**Total % extent**	33.7	51.8	79.8	57.7	83	83.1	66.7
	**Hip**[b]	**n clusters (t*)**	2 (4.3)	2 (4.3)	4 (4.2)	2 (4.7)	4 (4.5)	4 (4.2)	4 (4.3)
		**% extent (range)**	6.4 (27.9 - 34.3)<br>6.5 (46.6 - 53.1)	17.3 (20.4 - 37.7)<br>6.9 (46.9 - 53.8)	10.3 (26 - 36.3)<br>8.6 (46.1 - 54.7)<br>4 (75.2 - 79.2)<br>8.1 (91.9 - 100)	11.7 (24.7 - 36.4)<br>4.7 (95.3 - 100)	4.9 (49.2 - 54.1)<br>5.3 (63.9 - 69.2)<br>6.9 (73.8 - 80.7)<br>3.4 (95.5 - 98.9)	16.7 (24.2 - 40.9)<br>5.1 (48.8 - 53.9)<br>7.7 (61.2 - 68.9)<br>7.5 (73.7 - 81.2)	12.1 (25.9 - 38)<br>6.5 (48 - 54.5)<br>6.2 (61.6 - 67.8)<br>6.3 (73.5 - 79.8)
		**Total % extent**	12.9	24.2	31	16.4	20.5	37	25.1
	**Knee**[b]	**n clusters (t*)**	-	1 (.5)	5 (4.2)	2 (5.2)	3 (5.0)	6 (4.2)	3 (4.8)
		**% extent (range)**		3.5 (21.9 - 25.4)	3.7 (0 - 3.7)<br>7.9 (19 - 26.9)<br>4.2 (65.1 - 69.3)<br>6.1 (74 - 80.1)<br>6.8 (84.9 - 91.7)	6.5 (17 - 23.5)<br>4.4 (86.8 - 91.2)	5.3 (42.7 - 48)<br>7.9 (63.2 - 71.2)<br>6.7 (86 - 92.7)	5.8 (7.2 - 13)<br>5.7 (33.7 - 39.4)<br>4.6 (43.9 - 48.5)<br>10.2 (61.8 - 72)<br>6.1 (74.6 - 80.7)<br>7.2 (85.5 - 92.7)	4.8 (31.7 - 36.5)<br>8.4 (61.6 - 70)<br>6.8 (87.6 - 94.4)
		**Total % extent**		3.5	28.7	10.9	19.9	39.6	20
	**Ankle**[b]	**n clusters (t*)**	2 (4.3)	3 (4.44)	5 (4.2)	3 (4.6)	3 (4.7)	4 (4.1)	3 (4.6)
		**% extent (range)**	9.1 (47.2 - 56.3)<br>10.7 (67.2 - 77.9)	10.3 (4.9 - 15.2)<br>7.2 (19.7 - 26.9)<br>9.3 (46.6 - 55.9)	7.1 (4.2 - 11.3)<br>7.2 (17.7 - 24.9)<br>5.3 (35.1 - 40.4)<br>11.5 (46.1 - 57.6)<br>5.9 (64.9 - 70.8)	5.6 (3.6 - 9.2)<br>11 (14.8 - 25.8)<br>8.5 (49.2 - 57.7)	9.1 (0.9 - 10)<br>26.5 (15.6 - 42.1)<br>8.4 (49.6 - 58)	8.8 (3.3 - 12.1)<br>20.6 (18.8 - 39.4)<br>10.5 (47.3 - 57.8)<br>9.7 (66.5 - 76.2)	7.2 (5.7 - 12.9)<br>9.5 (47.3 - 56.8)<br>5.6 (66.8 - 72.4)
		**Total % extent**	19.8	26.8	37	25.1	44	49.6	22.3

[a]$\alpha = 0.007$; [b]$\alpha = 0.002$.
all $p \leq 0.001$.
All statistically significant clusters of segments or joints (up to 60% of the gait cycle) were additionally judged based on whether they identified differences larger than the standard errors of measurement (SEM), suggested by Everaert et al. (2024) [2]. Stance phase clusters highlighted in blue exceeded the respective SEM for more than 80% of their duration.
Vector: component consisting of the combination of the individual sagittal plane motions of the pelvis, hip, knee, and ankle joints; n clusters: number of identified suprathreshold clusters; T2*/t*: critical thresholds needed to reject the null hypothesis; % extent (range): extent of the identified suprathreshold cluster (start and end points of the identified cluster).

Table S4

Hypothesis 1b: Content validity - Coronal and transverse plane kinematic deviations between typically developing gait (N=56) and children with spastic cerebral palsy (N=270).

			Mild deviations (n=33)	Dropfoot (n=28)	Genu recurvatum (n=51)	True equinus (n=17)	Jump gait (n=28)	Apparent equinus (n=71)	Crouch gait (n=42)
Coronal plane	Vector[a]	n clusters (T2*)	3 (15.9)	1 (15.9)	-	-	2 (16.6)	1 (15.1)	1 (14.9)
		% extent (range)	7.6 (5.1 - 12.7)*	54.5 (22 - 76.5)**			20.4 (52.7 - 73.1)**	25.9 (45.6 - 71.5)**	26.5 (45.2 - 71.7)**
			18.1 (27.2 - 45.3)*				8.5 (84.3 - 92.8)*		
			17.7 (51 - 68.7)**						
		Total % extent	43.4	54.5			28.9	25.9	
	Pelvis[b]	n clusters (t*)	2 (3.7)	2 (3.8)			-	-	-
		% extent (range)	15.2 (0 - 15.2)**	20.5 (26.7 - 47.2)**					
			15.4 (26.5 - 41.9)**	22.8 (54.6 - 77.4)**					
		Total % extent	30.6	43.3					
	Hip[b]	n clusters (t*)	-	-			1 (3.9)	1 (3.6)	1 (3.5)
		% extent (range)					21.6 (51.8 - 73.4)**	7.2 (52.7 - 59.9)**	24.4 (47.5 - 71.9)**
		Total % extent					21.6	7.2	24.4

Table S4 ***(continued)***

			Mild deviations (n=33)	Dropfoot (n=28)	Genu recurvatum (n=51)	True equinus (n=17)	Jump gait (n=28)	Apparent equinus (n=71)	Crouch gait (n=42)
**Transverse plane**	**Vector[a]**	**n clusters (T2*)**	-	2 (19.8)	3 (18.1)	3 (24.8)	3 (19.9)	2 (17.5)	3 (18.0)
		**% extent (range)**		42.6 (22.8 - 65.4)**	4.5 (0 - 4.5)**	6.5 (0 - 6.5)**	4.6 (0 - 4.6)**	19.5 (44.2 - 63.7)**	8.7 (55.1 - 63.8)*
				27.9 (72.1 - 100)**	35.4 (24.1 - 59.5)**	21.1 (11.6 - 32.7)**	66.7 (17.2 - 83.9)**	6.6 (93.4 - 100)**	7.6 (80.2 - 87.8)*
					7.7 (92.3 - 100)**	12 (88 -100)**	8.7 (91.3 - 100)**		7.4 (92.6 - 100)**
		**Total % extent**		70.5	47.6	39.6	80	26.1	
	**Pelvis[c]**	**n clusters (t*)**		1 (4.2)	1 (3.6)	-	1 (3.8)	1 (3.6)	1 (3.8)
		**% extent (range)**		39.8 (24.4 - 64.2)**	19.2 (38.5 - 57.7)**		15.6 (27 - 42.6)**	15 (45 - 60)**	10.6 (83.1 - 93.7)**
		**Total % extent**		39.8	19.2		15.6	15	10.6
	**Hip[c]**	**n clusters (t*)**		-	2 (3.8)	3 (3.9)	2 (4.0)	1 (3.6)	1 (3.7)
		**% extent (range)**			4.8 (0 - 4.8)**	8.6 (0 - 8.6)**	4.4 (0 - 4.4)**	6.6 (93.4 - 100)**	6.3 (93.7 - 100)**
					7.3 (92.7 - 100)**	12.7 (44.8 - 57.5)**	8.1 (91.9 - 100)**		
						10.6 (89.4 - 100)**			
		**Total % extent**			12.1	31.9	12.5		6.3
	**Foot[c]**	**n clusters (t*)**		1 (3.9)	-	4 (3.4)	2 (3.5)	-	-
		**% extent (range)**		14.2 (75.4 - 89.6)**		4.8 (0 - 4.8)**	4.1 (0 - 4.1)**		
						20.8 (26 - 46.8)**	68.8 (31.2 - 100)**		
						11.6 (74 - 85.6)**			
						12.3 (87.7 - 100)**			
		**Total % extent**				49.5	72.9		

[a]$\alpha = 0.007$; [b]$\alpha = 0.0035$; [c]$\alpha = 0.002$.

*$p \leq 0.01$; **$p \leq 0.001$.

All statistically significant clusters of segments or joints were additionally judged based on whether they identified differences larger than the standard errors of measurement (SEM) for the entire gait cycle, suggested by Everaert et al. (2024) [2]. All identified clusters exceeded the respective SEM for more than 80% of their duration.

Vector: component consisting of the combination of the individual coronal or transverse plane motions; n clusters: number of identified suprathreshold clusters; T2*/t*: critical thresholds needed to reject the null hypothesis; % extent (range): extent of the identified suprathreshold cluster (start and end points of the identified cluster).

Table S5

Hypothesis 2a: Construct validity – Kinematic (N=270) and kinetic (N=208) vectors' comparisons among all spastic cerebral palsy gait patterns.

**Sagittal plane kinematics**	**n clusters (X2*)**	1 (43.6)
	**% extent (range)**	100 (0 - 100)**
**Sagittal plane moments**	**n clusters (X2*)**	2 (41.1)
	**% extent (range)**	63.9 (0.5 - 64.4)**<br>34 (65.8 - 99.8)**
**Sagittal plane powers**	**n clusters (X2*)**	4 (42.0)
	**% extent (range)**	9.6 (0 - 9.6)**<br>46.6 (13 - 59.6)**<br>12 (63.4 - 75.4)**<br>13.8 (86.2 - 100)**
**Coronal plane kinematics**	**n clusters (X2*)**	1 (27.8)
	**% extent (range)**	67.7 (26.7 - 94.4)**
**Transverse plane kinematics**	**n clusters (X2*)**	2 (35.2)
	**% extent (range)**	56.4 (28.6 - 85)**<br>9.9 (90.1 - 100)*

$\alpha=0.05$

*p ≤ 0.05; **p ≤ 0.001

n clusters: number of identified suprathreshold clusters; X2*: critical thresholds needed to reject the null hypothesis; % extent (range): extent of the identified suprathreshold cluster (start and end points of the identified cluster).

Table S6

Hypothesis 2a: Construct validity - Sagittal plane kinematic (N=270) and kinetic (N=208) deviations: comparisons between neighboring gait patterns.

			**Mild deviations vs. Dropfoot**	**Dropfoot vs. Genu recurvatum**	**Genu recurvatum vs. True equinus**	**True equinus vs. Jump gait**	**Jump gait vs. Apparent equinus**	**Apparent equinus vs. Crouch gait**
**Kinematics**	**Vector[a]**	**n clusters (T2*)**	3 (23.3)	1 (22.8)	2 (28.2)	1 (25.5)	1 (23.8)	1 (120.8)
		**% extent (range)**	9.4 (0 - 9.4)**	74.8 (9.8 - 84.6)**	59.5 (0 - 59.5)**	44.9 (11.7 - 56.6)**	100 (0 - 100)**	100 (0 - 100)**
			4.2 (28.5 - 32.7)*		4.2 (95.8 - 100)*			
			36.6 (63.4 - 100)**					
		**Total % extent**	50.2	74.8	63.7	44.9	100	100
	**Pelvis[b]**	**n clusters (t*)**	-	1 (3.7)	-	-	-	-
		**% extent (range)**		12.8 (66.7 - 79.5)**				
		**Total % extent**		12.8				
	**Hip[b]**	**n clusters (t*)**	-	-	-	-	-	-
	**Knee[b]**	**n clusters (t*)**	-	1 (4.0)	1 (4.6)	2 (4.1)	2 (4.3)	2 (3.8)
		**% extent (range)**		64.1 (14.2 - 78.3)**	20.3 (32.5 - 52.8)**	9.8 (0 - 9.8)**	5.3 (0 - 5.3)**	32.4 (14.7 - 47.1)**
						47.2 (10.1 - 57.3)**	14 (86 - 100)**	6 (57 - 63)**
		**Total % extent**		64.1	20.3	57	19.3	38.4
	**Ankle[b]**	**n clusters (t*)**	3 (4.0)	-	1 (4.5)	-	1 (3.3)	1 (4.3)
		**% extent (range)**	3.6 (0 - 3.6)**		21.3 (26.1 - 47.4)**		83.9 (16.1 - 100)**	100 (0 - 100)**
			8.7 (27.6 - 36.3)**					
			39.8 (60.2 - 100)**		21.3			
		**Total % extent**	52.1				83.9	100

Table S6 *(continued)*

			Mild deviations vs. Dropfoot	Dropfoot vs. Genu rrecurvatum	Genu recurvatum vs. True equinus	True equinus vs. Jump gait	Jump gait vs. Apparent equinus	Apparent equinus vs. Crouch gait
**Moments**	**Vector**[a]	**n clusters (T2*)**	2 (23.7)	2 (22.9)	1 (29.5)	1 (29.6)	-	-
		**% extent (range)**	14.8 (10.8 - 25.6)**	36.8 (21.1 - 57.9)**	12.4 (34.3 - 46.7)**	13.5 (30.1 - 43.6)**		
			3.9 (81.6 - 85.5)**	4.5 (91.5 - 96)**				
		**Total % extent**	18.7	41.3	12.4	13.5		
	**Hip**[b]	**n clusters (t*)**	-	-	-	-	-	
	**Knee**[b]	**n clusters (t*)**	-	1 (4.2)	-	1 (4.5)	-	
		**% extent (range)**		14.8 (30 - 44.8)**		17.5 (29.2 - 46.8)**		
		**Total % extent**		14.8				
	**Ankle**[b]	**n clusters (t*)**	2 (4.2)	-	-	-	-	
		**% extent (range)**	17.5 (2.1 - 19.6)**					
			7 (81.8 - 88.8)**					
		**Total % extent**	24.5					
**Powers**	**Vector**[a]	**n clusters (T2*)**	1 (23.8)	1 (24.3)	-	-	1 (31.9)	-
		**% extent (range)**	4.2 (20.5 - 24.7)**	5.8 (52.3 - 58.1)**			4.5 (16.3 - 20.8)**	
		**Total % extent**	4.2	5.8			4.5	
	**Hip**[b]	**n clusters (t*)**	-	-			-	
	**Knee**[b]	**n clusters (t*)**	-	-			-	
	**Ankle**[b]	**n clusters (t*)**	-	1 (4.4)			1 (4.9)	
		**% extent (range)**		4.3 (52.2 - 56.5)**			6.6 (16.5 - 23.1)**	
		**Total % extent**		4.3			6.6	

[a]α=0.008; [b]α=0.002.

*$p \leq 0.01$; **$p \leq 0.001$.

All statistically significant clusters were additionally judged based on whether they identified differences larger than the standard errors of measurement (SEM) for the entire gait cycle, suggested by Everaert et al. (2024) [2] (for moments and powers clusters identified up to 60% of the gait cycle). All identified clusters exceeded the respective SEM for more than 80% of their duration.

Vector: component consisting of the combination of the individual sagittal plane motions of the pelvis, hip, knee, and ankle joints; n clusters: number of identified suprathreshold clusters; T2*/t*: critical thresholds needed to reject the null hypothesis; % extent (range): extent of the identified suprathreshold cluster (start and end points of the identified cluster).

Table S7

Hypothesis 2b: Differences in age - pairwise comparisons between gait patterns.

**Compared pattern**	**Mild deviations**			**Dropfoot**			**Genu recurvatum**			**True equinus**			**Jump gait**			**Apparent equinus**		
	**Mean ranks**		**p**	**Mean ranks**		**p**	**Mean ranks**		**p**	**Mean ranks**		**p**	**Mean ranks**		**p**	**Mean ranks**		**p**
**Dropfoot**	32.8	28.9	0.385															
**Genu recurvatum**	**56.8**	33.3	**<0.001**	**51.3**	33.8	**0.001**												
**True equinus**	**29.7**	17.4	**0.005**	26.2	17.7	0.035	34.3	35.0	0.899									
**Jump gait**	**39.3**	21.2	**<0.001**	**35.1**	21.9	**0.003**	40.7	38.8	0.724	24.2	22.3	0.631						
**Apparent equinus**	57.6	50.1	0.238	50.7	49.7	0.886	47.1	**71.9**	**<0.001**	31.8	47.5	0.022	34.3	**56.2**	**0.001**			
**Crouch gait**	36.3	39.3	0.568	31.6	38.1	0.191	35.0	**61.5**	**<0.001**	19.1	**34.4**	**0.002**	22.9	**43.9**	**<0.001**	52.9	64.0	0.080

In every comparison, the mean ranks of the gait pattern in the respective column are presented first, followed by the mean ranks of the gait pattern in the respective row.
α=0.007. Effect sizes (d) for the Mann-Whitney U comparisons were calculated at https://www.psychometrica.de/effect_size.html - section 11. For statistically significant differences between two scores, the p-values and the mean ranks of the highest score are indicated in bold. The retrieved effect sizes are depicted using the following coloring scheme: $0.5 \leq d < 0.8$ a medium-sized effect (grey) and $d \geq 0.8$ indicates a large effect (dark grey) [3].

Table S8
Hypothesis 2b: Differences in spasticity impairment scores - pairwise comparisons between gait patterns.

**Compared pattern**	**Scores**	**Mild deviations**			**Dropfoot**			**Genu recurvatum**			**True equinus**			**Jump gait**			**Apparent equinus**		
		**Mean ranks**		**p**	**Mean ranks**		**p**	**Mean ranks**		**p**	**Mean ranks**		**p**	**Mean ranks**		**p**	**Mean ranks**		**p**
**Dropfoot**	**Comp**	26.39	36.43	0.026															
	**Psoas**	29.30	33.00	0.337															
	**Hams**	30.71	31.34	0.884															
	**RF**	30.64	31.43	0.801															
	**Gas**	29.20	33.13	0.325															
	**Sol**	24.51	**38.64**	**0.001**															
**Genu recurvatum**	**Comp**	27.94	**51.92**	**<0.001**	30.63	**45.15**	**0.007**												
	**Psoas**	32.41	**49.03**	**0.001**	32.57	44.08	0.021												
	**Hams**	34.89	47.42	0.015	32.43	44.16	0.021												
	**RF**	34.58	**47.63**	**0.005**	32.36	44.20	0.011												
	**Gas**	32.88	**48.73**	**0.001**	34.66	42.93	0.096												
	**Sol**	31.20	**49.81**	**<0.001**	40.79	39.57	0.801												
**True equinus**	**Comp**	20.50	**35.21**	**0.001**	19.64	28.53	0.026	33.94	36.18	0.685									
	**Psoas**	22.12	32.06	0.010	20.43	27.24	0.065	34.37	34.88	0.922									
	**Hams**	24.17	28.09	0.342	21.79	25.00	0.396	35.70	30.91	0.358									
	**RF**	22.76	30.82	0.019	20.39	27.29	0.036	34.28	35.15	0.864									
	**Gas**	20.79	**34.65**	**<0.001**	19.63	28.56	0.019	32.34	40.97	0.094									
	**Sol**	20.58	**35.06**	**<0.001**	21.13	26.09	0.193	32.42	40.97	0.105									
**Jump gait**	**Comp**	18.21	**46.07**	**<0.001**	15.93	**41.07**	**<0.001**	32.23	**54.16**	**<0.001**	16.44	26.98	0.009						
	**Psoas**	20.86	**42.95**	**<0.001**	19.18	**37.82**	**<0.001**	34.65	**49.75**	**0.003**	17.94	26.07	0.033						
	**Hams**	21.12	**42.64**	**<0.001**	18.50	**38.50**	**<0.001**	32.82	**53.07**	**<0.001**	14.65	**28.07**	**<0.001**						
	**RF**	22.61	**40.89**	**<0.001**	20.11	**36.89**	**<0.001**	36.12	47.07	0.030	19.56	25.09	0.153						
	**Gas**	21.17	**42.59**	**<0.001**	20.88	**36.13**	**<0.001**	33.91	**51.09**	**0.001**	20.59	24.46	0.311						
	**Sol**	21.56	**42.13**	**<0.001**	24.27	32.73	0.039	35.41	48.36	0.009	21.94	23.64	0.659						
**Apparent equinus**	**Comp**	33.47	**61.35**	**<0.001**	36.39	**55.37**	**0.003**	60.92	61.92	0.878	45.65	44.23	0.836	**68.71**	42.62	**<0.001**			
	**Psoas**	39.03	**58.76**	**0.001**	40.00	53.94	0.020	61.55	61.46	0.989	44.97	44.39	0.929	**62.93**	44.90	**0.003**			
	**Hams**	39.39	**58.59**	**0.002**	36.98	**55.13**	**0.003**	57.62	64.29	0.279	36.41	46.44	0.128	**63.75**	44.58	**0.002**			
	**RF**	40.03	**58.30**	**0.001**	38.00	**54.73**	**0.003**	60.80	62.00	0.841	44.32	44.54	0.973	59.73	46.16	0.025			
	**Gas**	41.45	**57.63**	**0.005**	44.30	52.25	0.184	62.75	60.61	0.722	53.85	42.26	0.075	**65.25**	43.99	**<0.001**			
	**Sol**	38.62	**58.95**	**<0.001**	53.73	48.53	0.361	64.06	59.66	0.443	56.09	41.73	0.022	**64.55**	44.26	**0.001**			

Table S8 ***(continued)***

Compared pattern	Scores	Mild deviations			Dropfoot			Genu recurvatum			True equinus			Jump gait			Apparent equinus		
		Mean ranks		p	Mean ranks		p	Mean ranks		p	Mean ranks		p	Mean ranks		p	Mean ranks		p
Crouch gait	**Comp**	26.73	**46.86**	**<0.001**	28.09	40.44	0.012	46.16	48.02	0.739	30.35	29.86	0.920	**45.07**	29.12	**0.001**	56.61	57.67	0.867
	**Psoas**	28.32	**45.61**	**<0.001**	27.64	**40.74**	**0.005**	44.97	49.46	0.399	28.21	30.73	0.593	40.88	31.92	0.059	55.01	60.37	0.377
	**Hams**	31.05	43.46	0.011	28.80	39.96	0.018	45.45	48.88	0.521	25.97	31.63	0.228	**43.68**	30.05	**0.004**	57.43	56.27	0.849
	**RF**	30.03	**44.26**	**0.001**	27.82	**40.62**	**0.003**	45.34	49.01	0.479	28.82	30.48	0.718	39.54	32.81	0.156	55.89	58.88	0.613
	**Gas**	33.12	41.83	0.052	33.75	36.67	0.520	49.79	43.61	0.229	37.41	27.00	0.024	**46.38**	28.25	**<0.001**	58.85	53.87	0.400
	**Sol**	32.70	42.17	0.038	39.98	32.51	0.097	50.87	42.30	0.089	38.50	27.00	0.010	**45.71**	28.69	**<0.001**	59.51	52.76	0.235

In every comparison, the mean ranks of the gait pattern in the respective column are presented first, followed by the mean ranks of the gait pattern in the respective row.
$\alpha=0.007$. Effect sizes (d) for the Mann-Whitney U comparisons were calculated at https://www.psychometrica.de/effect_size.html - section 11. For statistically significant differences between two scores, the p-values and the mean ranks of the highest score are indicated in bold. The retrieved effect sizes are depicted using the following coloring scheme: $0.5 \le d < 0.8$ a medium-sized effect (grey) and $d \ge 0.8$ indicates a large effect (dark grey) [3].
Comp: composite spasticity score; P: psoas; Hams: hamstrings; RF: rectus femoris; Gas: gastrocnemius; Sol: soleus.

Table S9

Hypothesis 2b: Differences in strength impairment scores - pairwise comparisons between gait patterns.

**Compared pattern**	**Scores**	**Mild deviations**			**Dropfoot**			**Genu recurvatum**			**True equinus**			**Jump gait**			**Apparent equinus**		
		**Mean ranks**		**p**	**Mean ranks**		**p**	**Mean ranks**		**p**	**Mean ranks**		**p**	**Mean ranks**		**p**	**Mean ranks**		**p**
**Dropfoot**	**Comp**	36.06	25.04	0.015															
	**Hfl**	33.21	28.39	0.222															
	**Hext**	32.74	28.95	0.353															
	**Kfl**	32.00	29.82	0.578															
	**Kext**	34.03	27.43	0.086															
	**DF90**	**37.86**	22.91	**<0.001**															
	**DF0**	**37.94**	22.82	**<0.001**															
	**PF**	34.67	26.68	0.053															
**Genu recurvatum**	**Comp**	**62.23**	29.74	**<0.001**	**54.50**	32.04	**<0.001**												
	**Hfl**	**54.83**	34.52	**<0.001**	**48.71**	35.22	**0.003**												
	**Hext**	**55.15**	34.31	**<0.001**	**50.95**	33.99	**0.001**												
	**Kfl**	**55.42**	34.14	**<0.001**	**52.57**	33.10	**<0.001**												
	**Kext**	**52.77**	35.85	**<0.001**	45.82	36.80	0.028												
	**DF90**	**60.58**	30.80	**<0.001**	48.18	35.51	0.010												
	**DF0**	**61.91**	29.94	**<0.001**	**49.43**	34.82	**0.002**												
	**PF**	**57.68**	32.68	**<0.001**	**50.09**	34.46	**0.002**												
**True equinus**	**Comp**	**32.73**	11.47	**<0.001**	**28.57**	13.82	**<0.001**	35.65	31.06	0.405									
	**Hfl**	**31.15**	14.53	**<0.001**	**27.68**	15.29	**<0.001**	36.60	28.21	0.070									
	**Hext**	**29.38**	17.97	**0.005**	26.29	17.59	0.016	34.00	36.00	0.687									
	**Kfl**	**30.70**	15.41	**<0.001**	**28.18**	14.47	**<0.001**	35.62	31.15	0.364									
	**Kext**	**30.14**	16.50	**0.001**	**26.61**	17.06	**0.004**	36.32	29.03	0.109									
	**DF90**	**31.83**	13.21	**<0.001**	26.46	17.29	0.014	35.28	32.15	0.540									
	**DF0**	**32.15**	12.59	**<0.001**	**26.75**	16.82	**0.007**	35.18	32.47	0.585									
	**PF**	**30.73**	15.35	**<0.001**	26.38	17.44	0.015	34.27	35.18	0.856									

Table S9 ***(continued)***

Compared pattern	Scores	Mild deviations			Dropfoot			Genu recurvatum			True equinus			Jump gait			Apparent equinus		
		**Mean ranks**		**p**	**Mean ranks**		**p**	**Mean ranks**		**p**	**Mean ranks**		**p**	**Mean ranks**		**p**	**Mean ranks**		**p**
**Jump gait**	**Comp**	**42.94**	16.93	**<0.001**	**38.89**	18.11	**<0.001**	44.41	31.96	0.020	25.59	21.43	0.299						
	**Hfl**	**39.00**	21.57	**<0.001**	**34.86**	22.14	**0.001**	42.16	36.07	0.187	21.88	23.68	0.614						
	**Hext**	**39.64**	20.82	**<0.001**	**36.48**	20.52	**<0.001**	41.92	36.50	0.260	25.88	21.25	0.189						
	**Kfl**	**40.65**	19.63	**<0.001**	**38.36**	18.64	**<0.001**	43.80	33.07	0.025	25.03	21.77	0.346						
	**Kext**	**39.50**	20.98	**<0.001**	**35.71**	21.29	**<0.001**	**44.56**	31.70	**0.006**	24.74	21.95	0.443						
	**DF90**	**42.35**	17.63	**<0.001**	**35.32**	21.68	**0.001**	42.49	35.46	0.155	24.18	22.29	0.611						
	**DF0**	**42.20**	17.80	**<0.001**	**35.39**	21.61	**0.001**	42.32	35.77	0.177	24.35	22.18	0.558						
	**PF**	**40.12**	20.25	**<0.001**	**35.04**	21.96	**0.001**	41.05	38.09	0.538	24.50	22.09	0.498						
**Apparent equinus**	**Comp**	**75.38**	41.87	**<0.001**	**63.52**	44.67	**0.003**	56.22	65.30	0.159	35.38	46.68	0.100	36.38	**55.37**	**0.003**			
	**Hfl**	**67.42**	45.56	**<0.001**	59.75	46.15	0.010	59.12	63.21	0.438	33.68	47.09	0.020	42.50	52.96	0.055			
	**Hext**	**66.59**	45.95	**<0.001**	**60.95**	45.68	**0.007**	56.60	65.02	0.149	41.53	45.21	0.549	39.98	53.95	0.015			
	**Kfl**	**66.68**	45.91	**<0.001**	**63.25**	44.77	**0.001**	57.60	64.30	0.251	36.12	46.51	0.096	37.09	**55.09**	**0.002**			
	**Kext**	**68.70**	44.97	**<0.001**	**60.84**	45.73	**0.005**	64.78	59.14	0.296	40.50	45.46	0.408	42.04	53.14	0.051			
	**DF90**	**73.79**	42.61	**<0.001**	55.98	47.64	0.150	56.27	65.25	0.130	36.15	46.50	0.102	38.71	**54.45**	**0.007**			
	**DF0**	**72.08**	43.40	**<0.001**	54.36	48.28	0.298	53.62	67.16	0.022	34.32	46.94	0.048	37.48	**54.94**	**0.003**			
	**PF**	**68.94**	44.86	**<0.001**	59.13	46.40	0.036	58.29	63.80	0.366	41.62	45.19	0.584	44.55	52.15	0.209			
**Crouch gait**	**Comp**	**54.09**	25.36	**<0.001**	**47.39**	27.57	**<0.001**	47.13	46.85	0.960	27.24	31.12	0.428	29.11	39.76	0.031	60.15	51.67	0.181
	**Hfl**	**48.29**	29.92	**<0.001**	**42.93**	30.55	**0.003**	47.54	46.35	0.798	25.56	31.80	0.142	32.93	37.21	0.323	58.88	53.82	0.338
	**Hext**	**48.58**	29.69	**<0.001**	**44.66**	29.39	**0.001**	47.72	46.13	0.758	32.06	29.17	0.524	33.68	36.71	0.503	60.41	51.24	0.113
	**Kfl**	**48.92**	29.42	**<0.001**	**46.21**	28.36	**<0.001**	48.08	45.69	0.636	28.44	30.63	0.622	31.14	38.40	0.098	60.22	51.56	0.135
	**Kext**	**48.44**	29.80	**<0.001**	**42.88**	30.58	**0.002**	49.72	43.70	0.188	28.12	30.76	0.532	31.09	38.44	0.094	57.63	55.94	0.757
	**DF90**	**49.38**	29.06	**<0.001**	37.75	34.00	0.408	42.85	52.04	0.078	24.15	32.37	0.075	27.93	**40.55**	**0.006**	55.85	58.95	0.595
	**DF0**	**51.59**	27.32	**<0.001**	39.61	32.76	0.114	43.15	51.68	0.084	24.47	32.24	0.077	28.54	40.14	0.009	58.18	55.00	0.582
	**PF**	**50.68**	28.04	**<0.001**	**43.80**	29.96	**0.002**	46.83	47.20	0.942	30.35	29.86	0.911	33.73	36.68	0.508	58.87	53.83	0.401

In every comparison, the mean ranks of the gait pattern in the respective column are presented first, followed by the mean ranks of the gait pattern in the respective row.
α=0.007. Effect sizes (d) for the Mann-Whitney U comparisons were calculated at https://www.psychometrica.de/effect_size.html - section 11. For statistically significant differences between two scores, the p-values and the mean ranks of the highest score are indicated in bold. The retrieved effect sizes are depicted using the following coloring scheme: $0.2 \le d < 0.5$ represents a small effect size (light grey); $0.5 \le d < 0.8$ a medium-sized effect (darker grey) and $d \ge 0.8$ indicates a large effect (dark grey) [3].
Comp: composite strength score; HfL: hip flexors; Hext: hip extensors; Kfl: knee flexors; Kext: knee extensors; DF90: ankle dorsiflexors with the knee in 90° flexion; DF0: ankle dorsiflexors with the knee extended; PF: plantarflexors.

Table S10

Hypothesis 2b: Differences in selectivity impairment scores - pairwise comparisons between gait patterns.

Compared pattern	Scores	Mild deviations			Dropfoot			Genu recurvatum			True equinus			Jump gait			Apparent equinus		
		Mean ranks		p	Mean ranks		p	Mean ranks		p	Mean ranks		p	Mean ranks		p	Mean ranks		p
**Dropfoot**	**Comp**	**38.09**	22.64	**0.001**															
	**Hfl**	32.50	29.23	0.05															
	**Hext**	31.88	29.96	0.529															
	**Kfl**	32.73	28.96	0.179															
	**Kext**	31.65	30.23	0.513															
	**DF90**	**37.30**	23.57	**0.001**															
	**DF0**	**39.03**	21.54	**<0.001**															
	**PF**	34.70	26.64	0.046															
**Genu recurvatum**	**Comp**	**62.71**	29.42	<0.001	**53.88**	32.38	<0.001												
	**Hfl**	**50.50**	37.32	<0.001	45.32	37.08	0.040												
	**Hext**	**53.67**	35.27	<0.001	**49.79**	34.63	**0.002**												
	**Kfl**	**54.95**	34.44	<0.001	**49.68**	34.69	**0.002**												
	**Kext**	**51.55**	36.65	<0.001	**47.93**	35.65	**0.005**												
	**DF90**	**60.35**	30.95	<0.001	48.57	35.29	0.010												
	**DF0**	**62.52**	29.55	<0.001	47.57	35.84	0.022												
	**PF**	**57.65**	32.70	<0.001	**51.57**	33.65	<0.001												
**True equinus**	**Comp**	**32.80**	11.32	<0.001	**28.23**	14.38	**0.001**	34.97	34.97	0.733									
	**Hfl**	**27.50**	21.62	**0.004**	24.14	21.12	0.234	33.95	33.95	0.617									
	**Hext**	28.36	19.94	0.011	25.39	19.06	0.053	33.63	33.63	0.490									
	**Kfl**	**30.32**	14.79	<0.001	**26.89**	16.59	**0.003**	35.15	35.15	0.607									
	**Kext**	**31.02**	14.79	<0.001	**28.14**	14.53	<0.001	36.85	36.85	0.055									
	**DF90**	**32.11**	12.68	<0.001	26.61	17.06	0.013	35.39	35.39	0.500									
	**DF0**	**32.83**	11.26	<0.001	26.45	17.32	0.017	35.27	35.27	0.556									
	**PF**	**30.30**	16.18	<0.001	26.21	17.71	0.015	33.63	33.63	0.512									

Table S10 ***(continued)***

Compared pattern	Scores	Mild deviations			Dropfoot			Genu recurvatum			True equinus			Jump gait			Apparent equinus		
		Mean ranks		p	Mean ranks		p	Mean ranks		p	Mean ranks		p	Mean ranks		p	Mean ranks		p
**Jump gait**	**Comp**	**43.62**	16.13	**<0.001**	**39.16**	17.84	**<0.001**	44.78	31.29	0.012	27.26	20.41	0.088						
	**Hfl**	**37.00**	23.93	**<0.001**	**33.00**	24.00	**0.007**	41.49	37.29	0.349	25.35	21.57	0.262						
	**Hext**	**38.91**	21.68	**<0.001**	**35.75**	21.25	**<0.001**	42.06	36.25	0.245	26.50	20.88	0.135						
	**Kfl**	**42.50**	17.45	**<0.001**	**38.64**	18.36	**<0.001**	**44.89**	31.09	**0.005**	26.85	20.66	0.077						
	**Kext**	**40.95**	19.27	**<0.001**	**38.04**	18.96	**<0.001**	**46.21**	28.70	**<0.001**	26.56	20.84	0.125						
	**DF90**	**43.08**	16.77	**<0.001**	**36.64**	20.36	**<0.001**	43.74	33.20	0.038	25.18	21.68	0.363						
	**DF0**	**43.09**	16.75	**<0.001**	**35.68**	21.32	**0.001**	43.41	33.79	0.063	25.56	21.45	0.286						
	**PF**	**40.80**	19.45	**<0.001**	**36.68**	20.32	**<0.001**	41.38	37.48	0.451	26.06	21.14	0.203						
**Apparent equinus**	**Comp**	**75.55**	41.79	**<0.001**	60.64	45.80	0.019	55.35	65.92	0.102	36.62	46.39	0.154	34.46	**56.13**	**0.001**			
	**Hfl**	**61.00**	48.55	**0.002**	54.80	48.11	0.134	59.03	63.27	0.397	44.44	44.51	0.989	43.71	52.48	0.084			
	**Hext**	**62.03**	48.07	**0.007**	57.68	46.97	0.047	56.63	65.00	0.154	43.15	44.82	0.783	40.68	53.68	0.026			
	**Kfl**	**64.64**	46.86	**0.001**	58.25	46.75	0.034	56.82	64.86	0.169	37.12	46.27	0.139	33.38	**56.56**	**<0.001**			
	**Kext**	**66.06**	46.20	**<0.001**	**62.07**	45.24	**0.002**	62.94	60.46	0.664	36.53	46.41	0.110	36.02	**55.51**	**0.001**			
	**DF90**	**71.73**	43.56	**<0.001**	54.36	48.28	0.318	54.33	66.65	0.047	34.32	46.94	0.054	34.20	**56.23**	**<0.001**			
	**DF0**	**76.59**	41.30	**<0.001**	53.41	48.65	0.425	54.32	66.65	0.043	33.62	47.11	0.036	35.05	**55.89**	**0.001**			
	**PF**	**67.33**	45.61	**<0.001**	58.14	46.79	0.060	55.98	65.46	0.131	41.35	45.25	0.555	41.02	53.54	0.043			
**Crouch gait**	**Comp**	**53.20**	26.06	**<0.001**	**45.54**	28.81	**0.001**	46.62	47.46	0.880	28.56	30.58	0.680	29.05	39.80	0.030	60.16	51.65	0.180
	**Hfl**	**46.50**	31.32	**<0.001**	**41.91**	31.23	**0.006**	49.23	44.30	0.295	33.44	28.61	0.245	35.64	35.40	0.956	60.56	50.98	0.061
	**Hext**	**45.23**	32.32	**0.002**	41.79	31.31	0.013	46.58	47.51	0.858	31.68	29.32	0.600	33.00	37.17	0.371	59.17	53.33	0.303
	**Kfl**	**50.59**	28.11	**<0.001**	**46.18**	28.38	**<0.001**	50.66	42.56	0.120	32.12	29.14	0.518	32.48	37.51	0.270	**62.99**	46.88	**0.006**
	**Kext**	**49.47**	28.99	**<0.001**	**46.18**	28.38	**<0.001**	51.79	41.18	0.037	29.79	30.08	0.949	30.82	38.62	0.095	60.84	50.51	0.074
	**DF90**	**48.71**	29.58	**<0.001**	37.07	34.45	0.576	41.66	53.49	0.028	22.88	32.88	0.032	25.04	**42.48**	**<0.001**	55.99	58.70	0.654
	**DF0**	**50.26**	28.37	**<0.001**	35.91	35.23	0.883	41.50	53.68	0.022	22.56	33.01	0.025	25.91	**41.89**	**0.001**	55.28	59.90	0.433
	**PF**	**49.30**	29.12	**<0.001**	**43.38**	30.25	**0.005**	45.31	49.05	0.491	30.38	29.85	0.909	31.61	38.10	0.174	58.87	53.83	0.412

In every comparison, the mean ranks of the gait pattern in the respective column are presented first, followed by the mean ranks of the gait pattern in the respective row.

α=0.007. Effect sizes (d) for the Mann-Whitney U comparisons were calculated at https://www.psychometrica.de/effect_size.html - section 11. For statistically significant differences between two scores, the p-values and the mean ranks of the highest score are indicated in bold. The retrieved effect sizes are depicted using the following coloring scheme: $0.2 \leq d < 0.5$ represents a small effect size (light grey); $0.5 \leq d < 0.8$ a medium-sized effect (darker grey) and $d \geq 0.8$ indicates a large effect (dark grey) [3].

Comp: composite selectivity score; HfL: hip flexors; Hext: hip extensors; Kfl: knee flexors; Kext: knee extensors; DF90: ankle dorsiflexors with the knee in 90° flexion; DF0: ankle dorsiflexors with the knee extended; PF: plantarflexors.

Table S11

Hypothesis 2b: Differences in pROM impairment scores - pairwise comparisons between gait patterns.

Compared pattern	Scores	Mild deviations			Dropfoot			Genu recurvatum			True equinus			Jump gait			Apparent equinus		
		Mean ranks		p	Mean ranks		p	Mean ranks		p	Mean ranks		p	Mean ranks		p	Mean ranks		p
**Dropfoot**	**Comp**	28.00	34.54	0.138															
	**Hext**	29.79	32.43	0.262															
	**Upopl**	30.61	31.46	0.833															
	**Bpopl**	32.03	29.79	0.596															
	**DF90**	26.24	36.61	0.012															
	**DF0**	27.58	35.04	0.059															
**Genu recurvatum**	**Comp**	41.94	42.86	0.862	44.30	37.64	0.205												
	**Hext**	37.53	45.72	0.024	37.57	41.33	0.331												
	**Upopl**	41.05	43.44	0.632	39.18	40.45	0.796												
	**Bpopl**	44.85	40.98	0.445	40.54	39.71	0.868												
	**DF90**	42.59	42.44	0.976	**48.71**	35.22	**0.006**												
	**DF0**	41.71	43.01	0.781	45.45	37.01	0.071												
**True equinus**	**Comp**	22.85	30.65	0.064	22.05	24.56	0.515	32.26	41.21	0.098									
	**Hext**	23.05	30.26	0.009	21.50	25.47	0.175	33.82	36.53	0.535									
	**Upopl**	24.68	27.09	0.541	22.39	24.00	0.656	34.21	35.38	0.818									
	**Bpopl**	26.05	24.44	0.693	22.93	23.12	0.959	34.25	35.24	0.848									
	**DF90**	23.65	29.09	0.164	24.30	20.85	0.322	32.63	40.12	0.133									
	**DF0**	21.77	**32.74**	**0.004**	21.21	25.94	0.181	31.02	**44.94**	**0.004**									
**Jump gait**	**Comp**	25.56	37.41	0.008	25.34	31.66	0.138	35.43	48.32	0.015	21.32	24.02	0.496						
	**Hext**	24.79	**38.32**	**<0.001**	23.86	33.14	0.009	36.38	46.59	0.024	20.18	24.71	0.211						
	**Upopl**	24.91	**38.18**	**0.002**	22.54	**34.46**	**0.003**	34.88	**49.32**	**0.004**	18.06	26.00	0.034						
	**Bpopl**	28.79	33.61	0.260	25.21	31.79	0.107	36.52	46.34	0.052	19.88	24.89	0.185						
	**DF90**	32.21	29.57	0.537	33.48	23.52	0.012	41.17	37.88	0.513	26.74	20.73	0.111						
	**DF0**	29.18	33.14	0.327	29.89	27.11	0.473	38.59	42.57	0.403	27.06	20.54	0.073						
**Apparent equinus**	**Comp**	41.71	57.51	0.011	45.57	51.75	0.324	52.87	67.70	0.020	43.91	44.64	0.914	53.77	48.51	0.404			
	**Hext**	44.11	56.40	0.008	45.21	51.89	0.171	59.96	62.61	0.605	45.65	44.23	0.800	57.63	46.99	0.050			
	**Upopl**	41.26	**57.73**	**0.006**	39.25	54.24	0.012	52.58	67.91	0.012	36.26	46.47	0.113	54.00	48.42	0.348			
	**Bpopl**	46.12	55.46	0.114	40.75	53.65	0.030	51.57	**68.51**	**0.006**	35.71	46.61	0.089	49.52	50.19	0.910			
	**DF90**	49.80	53.75	0.500	58.75	46.55	0.036	58.70	63.51	0.420	48.88	43.45	0.389	44.95	51.99	0.238			
	**DF0**	46.77	55.16	0.138	52.27	48.99	0.530	56.82	64.86	0.163	54.03	42.22	0.056	49.04	50.38	0.816			

Table S11 *(continued)*

Compared pattern	Scores	Mild deviations			Dropfoot			Genu recurvatum			True equinus			Jump gait			Apparent equinus		
		**Mean ranks**		**p**	**Mean ranks**		**p**	**Mean ranks**		**p**	**Mean ranks**		**p**	**Mean ranks**		**p**	**Mean ranks**		**p**
**Crouch gait**	**Comp**	33.45	41.57	0.098	35.09	35.09	0.887	43.2	51.58	0.127	32.03	29.18	0.551	39.05	33.13	0.222	58.60	54.30	0.491
	**Hext**	31.64	**43.00**	**0.002**	31.21	31.21	0.064	44.35	50.21	0.196	28.68	30.54	0.657	37.91	33.89	0.357	55.20	60.04	0.356
	**Upopl**	29.26	**44.87**	**0.001**	27.02	**27.02**	**0.002**	39.81	**55.73**	**0.002**	23.18	32.76	0.033	36.71	34.69	0.654	55.62	59.33	0.528
	**Bpopl**	30.92	**43.56**	**0.007**	26.68	**26.68**	**0.001**	37.98	**57.95**	**<0.001**	21.50	33.44	0.008	31.88	37.92	0.174	53.49	62.94	0.102
	**DF90**	43.85	33.40	0.025	**47.55**	47.55	**<0.001**	52.75	40.01	0.014	**40.35**	25.81	**0.001**	38.55	33.46	0.275	**63.94**	45.27	**0.002**
	**DF0**	40.58	35.98	0.314	42.61	42.61	0.009	50.15	43.18	0.167	**40.53**	25.74	**0.001**	40.32	32.29	0.079	62.45	47.79	0.012

In every comparison, the mean ranks of the gait pattern in the respective column are presented first, followed by the mean ranks of the gait pattern in the respective row.
α=0.007. Effect sizes (d) for the Mann-Whitney U comparisons were calculated at https://www.psychometrica.de/effect_size.html - section 11. For statistically significant differences between two scores, the p-values and the mean ranks of the highest score are indicated in bold. The retrieved effect sizes are depicted using the following coloring scheme: $0.2 \leq d < 0.5$ represents a small effect size (light grey); $0.5 \leq d < 0.8$ a medium-sized effect (darker grey) and $d \geq 0.8$ indicates a large effect (dark grey) [3].
The bilateral popliteal angle refers to the true hamstrings length and is measured with the contralateral hip in flexion, while the unilateral popliteal angle is measured with the contralateral hip in extension, and may thus be influenced by the pelvic position and involvement of the psoas [4].
pROM: passive range of motion; Comp: composite spasticity score; Hext: hip extension; Upopl: unilateral popliteal angle; Bpopl: bilateral popliteal angle; DF90: ankle dorsiflexors with the knee in 90°; DF0: ankle dorsiflexors with the knee extended.

References

[1] E. Papageorgiou, A. Nieuwenhuys, I. Vandekerckhove, A. Van Campenhout, E. Ortibus, K. Desloovere, Systematic review on gait classifications in children with cerebral palsy: An update, Gait Posture. 69 (2019) 209–223. doi:10.1016/j.gaitpost.2019.01.038.

[2] L. Everaert, T. Dewit, C. Huenaerts, A. Van Campenhout, L. Labey, Repeatability of gait of children with spastic cerebral palsy in different walking conditions, J. Biomech. 176 (2024) 112301. doi:10.1016/j.jbiomech.2024.112301.

[3] J. Cohen, Statistical power and analysis for the behavioral sciences (2nd ed.), Hillsdale, Erlbaum, 1988.

[4] M. Mohamed Tageldeen, B. Salmeen Awad Salmeen, D. Nader M, B. Ahmed Samir, Assessment of the Relationship between Hamstring Tightness and Pelvic Tilt in Cerebral Palsy: Are We Overdoing Hamstring Releases in Children with Spastic Bilateral CP, GMFCS Level I-III?, J. Orthop. Surg. Tech. 3 (2020) 167–174. doi:10.36959/453/542.
